# Update on the *Neisseria* Macrophage Infectivity Potentiator-Like PPIase Protein

**DOI:** 10.3389/fcimb.2022.861489

**Published:** 2022-03-22

**Authors:** Myron Christodoulides

**Affiliations:** Neisseria Research Group, Molecular Microbiology, Academic School of Clinical and Experimental Sciences, University of Southampton Faculty of Medicine, Southampton, United Kingdom

**Keywords:** *Neisseria meningitidis*, *Neisseria gonorrhoeae*, Macrophage Infectivity Potentiator, vaccine, targeted treatments, commensal *Neisseriae*

## Abstract

*Neisseria* pathogens express a Macrophage Infectivity Potentiator Protein (MIP), which belongs to the FK506 binding protein (FKBP) family of proteins that exhibit peptidyl-prolyl cis/trans isomerase (PPIase) activity. *Neisseria* MIP proteins are potential candidates for inclusion into vaccines for gonorrhoea caused by *N. gonorrhoeae* infection, and meningitis/sepsis caused by *M. meningitidis* infection. *Neisseria* MIP proteins are also potential targets for directed drug treatments, although this remains relatively unexplored. In this mini-review, we provide an update into the vaccine potential of *Neisseria* MIP and the few published drug targeting studies, and explore further the diversity of this protein amongst both pathogenic and commensal *Neisseria* spp.

## Introduction

The genus *Neisseria* contains the pathogens *Neisseria gonorrhoeae* (Ng, gonococcus) and *Neisseria meningitidis* (Nm, meningocococus) that can infect humans, and several commensal species and species that can infect other mammals (Humbert and Christodoulides, 2019). The gonococcus is an obligate human pathogen, not part of the normal human urogenital tract microbiota, and causes the sexually transmitted disease (STD) gonorrhoea (Quillin and Seifert, 2018). There are ~87 million cases of gonorrhoea reported annually worldwide, with the highest burden of infection borne by people in the least developed and low-to-middle income countries (Rowley et al., 2019). This figure is certainly an underestimate due to unreported asymptomatic infections. Gonococci colonize primarily the mucosal epithelium of the male urethra, causing urethritis and a painful discharge, and the mucosal epithelium of the female endo/ectocervix, causing a mucopurulent cervicitis. Asymptomatic infections are common in both genders (Quillin and Seifert, 2018), but more serious for women, and in approximately 10-25% of untreated women, gonococci can ascend into the upper reproductive tract and induce a clinical syndrome of Pelvic Inflammatory Disease (PID). Patients with PID can suffer long-term and/or permanent sequelae, e.g. chronic pelvic pain, Fallopian tube damage, endometritis, ectopic pregnancy and infertility (Quillin and Seifert, 2018; Stevens and Criss, 2018). Atypical infections can occur at other anatomical sites, because of gonococci disseminating from the urogenital tract, or develop as primary infections due to the direct interaction of the pathogen (Humbert and Christodoulides, 2019). Co-infection with other STD pathogens *e.g.* Human Immunodeficiency Virus (HIV), *Treponema pallidum*, and Chlamydia, is not uncommon. Notably, gonococcal infection is known to promote HIV transmission and infection (Guvenc et al., 2020).

The meningococcus is a commensal organism present within the human nasopharyngeal microbiota that can behave as an opportunistic pathogen to cause Systemic Meningococcal Disease (SMD), manifested as meningitis and/or sepsis (Brandtzaeg and Van Deuren, 2012). Meningococcal infection has caused historically significant mortality and morbidity (Schoen et al., 2008), but the development and introduction of capsule polysaccharide (CPS)-conjugate vaccines and the protein-based Bexsero and Trumenba vaccines has dramatically reduced cases of infection worldwide (Christodoulides and Heckels, 2017; Rivero-Calle et al., 2019). SMD is wholly preventable by vaccination, but there are no vaccines to prevent gonorrhoea (Wetzler et al., 2016). Moreover, the successful control of gonorrhoea with antibiotics has been compromised, due to the ability of the gonococcus to rapidly develop resistance to every class of antibiotic introduced (Unemo et al., 2019). There is an urgent need for new antimicrobials for gonorrhoea and for candidate vaccines for human trials.

In this mini-review, our aim is to update our previous comprehensive review (Humbert et al., 2015), with published research on the vaccine and therapeutic target potential of the *Neisseria* Macrophage Infectivity Potentiator (MIP) protein, which belong to the FK506 binding protein (FKBP) family of proteins that exhibit peptidyl-prolyl cis/trans isomerase (PPIase) activity.

## Biology and Structure of *Neisseria* MIP

Gonococci and meningococci both produce FKBP-type MIP PPIases that play roles in the pathogen-host interaction and offer potential targets for vaccines and therapies. Expression of a surface-exposed Ng-MIP lipoprotein (NG1225 = NEIS1487 in the http://pubmlst.org/Neisseria/database) of molecular mass (*Mr*) of 30 kDa appeared to be important for gonococcal persistence within mouse and human macrophages *in vitro* and for protection from the bactericidal activity of immune effector cells (Leuzzi et al., 2005). Ng-MIP protein was not involved in gonococcal adherence, since no differences were observed between parent and a Ng-MIP mutant in adhering to macrophages and subsequent invasion. However, the Ng-MIP mutant did show reduced survival following infection, suggesting that the protein played a role in the intracellular survival of the pathogen (Leuzzi et al., 2005). Ng-MIP appears to be expressed during infection *in vivo* and is immunogenic, with sera from patients with urethritis or disseminated gonococcal infections able to recognize purified Ng-MIP (Leuzzi et al., 2005; Starnino et al., 2010). Ng-MIP was identified as a target in gonococci for protein-linked O-glycosylation (Vik et al., 2009). Nm-MIP (NMB1567 = NEIS1487) is a MIP-like PPIase (*Mr* ~29 kDa) that was identified by proteomics in high abundance in the meningococcal outer membrane (OM) and found to share homology with the *Legionella pneumophila* (Lp)-MIP (Williams et al., 2007). Expression of Nm-MIP protein was important for bacterial survival in the blood, with expression of the *nm-mip* gene upregulated in an *ex vivo* human whole-blood model infected with wild type *N. meningitidis* strain MC58 (Echenique-Rivera et al., 2011).

However, it does appear that many PPIases are encoded within the *Neisseria* chromosome, and the reader can refer to a previous review (Humbert et al., 2015) for further information on these predominantly cytosolic proteins, including a *Neisseria* FKBP-like PPIase encoded by gene *nmb0027* (Sampson and Gotschlich, 1992; McAllister and Stephens, 1993).

**Figure 1:**
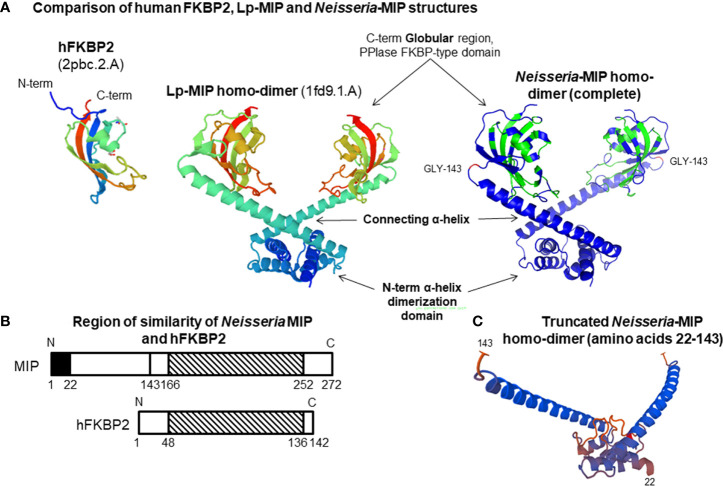
**(A)** Comparison of human FKBP2, *Legionella pneumophila* (Lp)-MIP and *Neisseria*-MIP structures. The *Neisseria*-MIP model is based on the crystal structure of Lp-MIP and consists of 3 domains as marked. Two monomers assemble to form a homo-dimer that associated with the bacterial OM. The structure of the human FKBP2 PPIase globular protein is shown for comparison. **(B)** Cartoon showing the overlapping region of similarity between *Neisseria* MIP and human FKBP2 protein. Similarity is within the striped C- terminal region. **(C)** Structure of the truncated MIP homo-dimer. The sequence of amino acids 22-143 located within the N-terminal region after removal of the globular regions that contain FKBP activity. Adapted from our figures published by Humbert et al. (2015) and Bielecka et al. (2015).

Based on the structure of Lp-MIP protein (Riboldi-Tunnicliffe et al., 2001; Ceymann et al., 2008), *Neisseria* MIP proteins exist as a homo-dimer in the bacterial OM, following cleavage of a N-terminal signal sequence peptide during transport across the cytoplasmic membrane. Each monomer of the homo-dimer can be structurally divided into i) a N-terminal α-helix that participates in a dimerization domain and is probably lipidated to allow anchoring of MIP into the OM (Bielecka et al., 2015); ii) a long connecting α-helix in the centre, ending with iii) a globular C-terminal domain containing the FKBP-type PPIase function (**Figure 1A**). BLAST sequence analyses demonstrated that the human (h)FKBP2 PPIase protein and Nm/Ng-MIP share ~ 48% amino acid similarity within the region located between amino acids 166 and 252 (Bielecka et al., 2015) (**Figure 1B**). Thus, *Neisseria* MIP is a partial molecular mimic of human FKBP protein, which may provide the pathogens with a survival advantage, possibly related to their survival within host cells and for immune avoidance.

## Update on Vaccine Potential of *Neisseria* MIP Proteins

*Neisseria* MIP proteins have several important attributes for inclusion into vaccines: the *mip* gene is present in all gonococci and meningococci examined thus far and the proteins are highly conserved, expressed by all strains (studied thus far), anchored in the OM and surface-exposed, and capable of inducing functional bactericidal antibodies (Leuzzi et al., 2005; Hung et al., 2011).

Ng-MIP and Nm-MIP are highly conserved proteins: in 2015, MIP allelic information and sequence diversity was examined in 5860 pathogenic *Neisseria* isolates in the http://pubmlst.org/Neisseria/database that had complete genome sequence information (Jolley and Maiden, 2010). For gonococci, the number of genomes that could be analyzed increased from 1217 in 2015 to 12805 in 2022, with the number of Ng-MIP allelic loci increasing from 25 to 66 and the number of non-redundant Ng-MIP amino acid sequences increasing from 16 to 32 (**Table 1** and **Supplementary Dataset 1**). The Clustal alignment of the non-redundant allele amino acid sequences is shown in **Supplementary Dataset 2** and the dendrogram in **Supplementary Figure 1**. The *ng-mip* gene was present in 100% of the genomes that could be analyzed using the PubMLST plug-in program. The dominant alleles expressed by gonococci were still alleles 10 and 35, with the number of isolates expressing allele 10 protein increasing from 49 to 64%, and the number of isolates expressing allele 35 decreasing from 35 to 22%. Despite these minor changes, expression of alleles 10 and 35 together still covered >84% of all gonococcal isolates examined, and >90% coverage would be obtained by including isolates expressing allele 140 protein (**Supplementary Table 1**).

**Table 1:** Updated total *Neisseria* Ng-MIP and Nm-MIP allelic information and sequence diversity.

MIP protein	Characteristic	Year	
		Accessed 2015	Accessed 2022
**Ng-MIP**	Number of gonococcal genomes	1217	12805
	Gene presence	1217/1217	12805/12805
	Number of allelic loci	25	66
	Non-redundant Ng-MIP amino acid sequences	16	32
	Majority Alleles	10 (49% of isolates) 35 (35% of isolates)	10 (64% of isolates) 35 (22% of isolates)
**Nm-MIP**	Number of meningococcal isolates	4643	26941
	Gene presence	4643/4643 (present across 15 sequence types)	26940/26941*
	Number of allelic loci	91	356 with isolates (additional 387 with no isolates)
	Non-redundant Nm-MIP amino acid sequences	44	149
	Majority Alleles	2 (49% of isolates; Type I Nm-MIP) 1 (29% of isolates; Type II Nm-MIP) 7 (10% of isolates; Type IV Nm-MIP)	2 (44% of isolates) 1 (28% of isolates) 7 (10% of isolates)

*Gene is absent in id 51598 (**Supplementary Table 1**).

For meningococci, the number of genomes that could be analyzed increased considerably from 4643 in 2015 to 26941 in 2022, with the number of Nm-MIP allelic loci increasing from 91 to 365 (with identifiable isolates) and the number of non-redundant Nm-MIP amino acid sequences increasing from 44 to 149 (**Table 1** and **Supplementary Dataset 3**). The Clustal alignment of the non-redundant allele amino acid sequences is shown in **Supplementary Dataset 4** and the dendrogram in **Supplementary Figure 2**. The *nm-mip* gene was present in all but one of the 26941 genomes that could be analyzed using the PubMLST plug-in program. The dominant alleles expressed by meningococci were still alleles 2, 1 and 7, with little significant change in the percentages of isolates expressing these alleles over the intervening years (**Table 1**). Taken together, expression of alleles 2, 1 and 7 proteins still covered >80% of all meningococcal isolates examined, and >90% coverage could be obtained by including the isolates expressing allele 22 and 13 proteins (**Supplementary Table 1**).

The vaccine potential of a recombinant (r)Nm-MIP protein was first shown by Hung et al. (Hung et al., 2011), who reported that antibodies raised to Type I rNm-MIP bound to the surface OM of meningococci and were bactericidal *via* a complement-dependent mechanism. Moreover, antibodies to Type 1 rNm-MIP had cross-strain bactericidal activity and could kill heterologous strains expressing Type II and III Nm-MIP proteins. Liposomal incorporation of rNm-MIP was preferred for inducing cross-strain bactericidal activity, whereas adsorption to Al(OH)_3_ was contra-indicated, suggesting that conformational refolding in the liposomal membrane is essential for inducing functional antibodies. Whether cross-protection extends to isolates expressing other allelic proteins has yet to be determined. Costoya and colleagues confirmed that liposomal presentation of a rMIP protein enhanced the bactericidal response (Costoya et al., 2017). Nm-MIP was also identified in a study of the meningococcal surface-ome and proteome (Tsolakos et al., 2014). MIP-specific antisera were shown to i) bind to whole meningococci in ELISA, ii) facilitate opsonophagocytosis and iii) deposit complement factors on the surface of meningococcal isolates of different serosubtypes.

Rational design of *Neisseria* MIPs for vaccines needs to consider i) the structure of the protein and ii) the potential amino acid sequence similarity with human PPIases. Clustal alignment of the amino acids of the whole protein encoded by Ng-MIP alleles 10, 35, 140 and Nm-MIP alleles 2, 1, 7, 22, 13, which provide >90% coverage of respective gonococcal and meningococcal isolates in the PubMLST *Neisseria* database, showed that there was overall ~96% sequence similarity (**Supplementary Datasets 2**, **4**). There was ~93% sequence similarity in the N-terminal amino acid region 22 – 143 amongst all these proteins (**Supplementary Datasets 2**, **4**). In order to bypass the homologous region of molecular mimicry of *Neisseria* MIP with hFKBP2 protein (amino acids 166 - 252), truncated proteins were generated by removing the globular domain that contains PPIase activity and sequence similarity. Significantly, a truncated rNm-MIP construct spanning amino acids 22 - 143 (**Figure 1C**) induced antibodies that killed strains expressing different Type Nm-MIP proteins and different CPS serogroups (Bielecka et al., 2015). Recently, we showed that antibodies raised to truncated rNm-MIP killed gonococci (Humbert and Christodoulides, 2018). These bactericidal antibodies do not cross-react with native pure hFKBP2 protein, whereas significant cross-reactivity was observed with antisera raised to full sequence rMIP proteins.

There is evidence that vaccination with meningococcal OM vesicle (OMV) vaccines can reduce the case incidence of gonorrhea. Anecdotal data from Cuba and Norway and the controlled retrospective case-control study in New Zealand suggested a decline in gonorrhea in the period immediately after use of meningococcal OMV vaccines (Petousis-Harris and Radcliff, 2019). The most robust data come from New Zealand, where a reduction in gonorrhea cases was observed after introduction of the MeNZB OMV vaccine. Vaccine effectiveness against gonorrhea in the population was estimated at 31% (95% Confidence Intervals 21–39) (Petousis-Harris et al., 2017). MenZB is a component of Bexsero, and a recent study in mice showed that immunization with Bexsero accelerated vaginal clearance of colonizing gonococci and that antibodies could recognize several gonococcal proteins (Leduc et al., 2020). One can speculate that antibodies to non-hFKBP2 regions of Nm-MIP in OMV contribute to protection against gonorrhoea. The MenZB strain is NZ98/254 (id 34542 in PubMLST/*Neisseria* database) and this strain expresses Allele 2 Type 1 Nm-MIP, the dominant allelic protein, which we know shares ~99% homology with Ng-MIP.

Another vaccine design approach was published by Gholami et al., who modelled *in silico* a chimeric antigen of Adhesin Complex Protein-MIP-PilQ (Gholami et al., 2015). Biophysical and biochemical properties and predictions of B cell, T cell and antigenic epitopes were made *in silico*, but disappointingly no *in vivo* immunological studies were reported. Moreover, the incorporation of full MIP protein into their design would be contra-indicated as shown above.

If the http://pubmlst.org/Neisseria/database is interrogated with analysis of gonococcal and meningococcal case reports for the recent year with the most complete available allele data, it is possible to estimate potential coverage of circulating isolates using MIP protein-based vaccines. For gonococci, the latest complete data are for 2019, in which there were 524 isolates of which 400 had Ng-MIP alleles identified. Consistent with the overall data shown in **Table 1**, the majority alleles for this most recent year were 10, 35 and 140 (**Supplementary Table 2**), which together accounted for 97% of all isolates. For meningococci, the latest complete data are for 2021, in which there were 198 isolates of which 166 had Nm-MIP alleles identified. Again, consistent with the overall data (**Table 1**), the majority alleles were 1 and 2, which together accounted for 80% of the isolates, with the inclusion of isolates expressing alleles 22 and 13 bringing this up to 90% coverage. Thus, vaccines containing 3 rNg-MIP proteins, or 3-5 rNm-MIP proteins, would be sufficient for almost total gonococcal and meningococcal isolate coverage, respectively. The observed cross-protection induced by any single recombinant *Neisseria* MIP protein (Hung et al., 2011; Bielecka et al., 2015; Humbert and Christodoulides, 2018) will reduce the number of antigens that are needed.

## Presence of MIP in Commensal *Neisseria*

The non-pathogenic *Neisseria* species comprise part of the commensal bacterial microbiota of the human and animal oropharynx, but these ‘apparently harmless’ commensals can produce infection in a wide variety of anatomical sites including the heart, nervous system (to cause meningitis), bloodstream (to cause septicaemia), respiratory tract, bone marrow, skin and genital tract. The distribution and location of these commensal *Neisseria* in and on the human body have been reviewed extensively elsewhere (Weyand, 2017; Humbert and Christodoulides, 2019). Commensal *Neisseria* in the upper respiratory tract (URT) can compete with meningococci for niche occupation and provide some protection against colonization. *Neisseria spp* have been identified in the URTs of many mammals, including dogs, cats, cattle and non-human primates. *N. mucosa* has been cultured even from the woodlouse, and from environmental water and sediment (Liu et al., 2015). Although animal to human transmission of *Neisseria spp* has been reported, e.g. through bite wounds and then causing infection, *Neisseria* spp are not zoonotic organisms in the strict sense of the term. Interestingly, the pathogenic *Neisseria* have been found in other mammals, with an intriguing recent report of gonococci isolated from the vaginal microbiome of giant pandas (Zhang et al., 2020).

With the increase in available genomes in the PubMLST/*Neisseria* database, it is possible to examine whether human commensal species and species that colonize other mammals also express MIP, and the diversity of their allelic expression. Of the 39 species additional to *N. gonorrhoeae* and *N. meningitidis*, and the two categories identified as *Neisseria sp* and Non-*Neisseria sp*, in the PubMLST/*Neisseria* database, MIP alleles were present in 13 species (*basseii, benedictiae, bergeri, blantyrii, cinerea, lactamica, maigaei, mucosa, oralis, polysaccharea, subflava, uirgultaei, viridiae*) and within the *Neisseria sp* category (**Supplementary Table 3**). A consideration for any *Neisseria* vaccine antigen is whether they are also potentially expressed by commensal *Neisseria spp*, since generated immune responses towards vaccine antigens may also target commensal species. This is particularly relevant to meningococci, as the 13 species isolates expressing MIP allele proteins have been isolated from throat swabs and are thus present in the oral cavity and nasopharynx. None of these 13 species isolates expressed MIP protein encoded by the dominant meningococcal Allele 1, 2, 7, 22 or 13. However, we did identify some minor allele proteins shared by these species with meningococci (**Supplementary Table 3**), notably allele 27 with *bergeri*, allele 113 with *cinerea*, alleles 9, 15, 58 and 324 with *lactamica* and alleles 88 and 229 with *polysaccharea.* No allele proteins were shared between gonococci and any of these other *Neisseria spp* (**Supplementary Table 3**). The one *Neisseria sp* category isolate IE21Nm045, id 95638, expressed Allele 10-encoded MIP protein and was isolated from the eye and was suggested to be a gonococcus by rMLST analysis.

Although commensal *Neisseria spp* do not share the major allele-encoded MIP proteins of the pathogens, the amino acid sequence similarities between the dominant gonococcal and meningococcal allele proteins and other allele proteins may still be high. To check this, we did a Clustal alignment of MIP proteins encoded by alleles 1, 2, 7, 22, 13 (dominant meningococcal) and 10, 34, 140 (dominant gonococcal) with MIP allele proteins from each of the other 13 species that are expressed by most isolates belonging to each of the species and excluding the ones mentioned in the previous paragraph (**Supplementary Dataset 5**). Examination of the vaccine-relevant N-terminal amino acid sequence 22-143 showed that there is significant similarity between the commensal and pathogen MIP protein sequences. Thus, within this sequence, Ng/Nm-MIP share ~ 93% sequence similarity with *benedictiae* and *cinerea* MIP, and 95-97% similarity with *basserii, bergeri, blantyrii, lactamica, maigaei, polysaccharea, viridae* and *uirgultaei* MIP. By contrast, Ng/Nm-MIP share only 69% similarity with *oralis* MIP, and 71% and 73% similarity with *subflava* and *mucosa* MIP respectively (**Supplementary Dataset 5**).

The structure of the MIP proteins from different *Neisseria spp* can be modelled on the crystal structure of Lp-MIP. As expected, there is remarkable conservation of structure between the MIP proteins (**Supplementary Figure 3**). Thus, there is the potential for antibodies generated to MIP, in particular Nm-MIP, to cross-react with MIP proteins expressed by another *Neisseria* spp. Commensal species occupying niches in the URT that bind anti-MIP antibodies could act inadvertently as decoys by adsorbing some of the available antibody pool and reducing immune clearance of colonizing pathogens. This would provide the pathogen with an advantage for colonization, although it is likely that other bacteria in the microbiota will still compete for these available niches.

## *Neisseria* MIP Proteins as Drug Targets

PPIase enzymes catalyze the slow *cis-trans* isomerization of proline imidic peptide bonds in oligopeptides and proteins and accelerate protein folding. PPIase activity has been shown for many bacterial MIP proteins, including Lp-MIP, Ng-MIP, *Chlamydia trachomatis-*MIP, *Coxiella burnetii*-MIP, *Burkholderia pseudomallei* (Bp)-MIP, *E.coli-*MIP, *Shewanella* sp. SIB1-MIP, and *Vibrio anguillarum*-MIP (Fischer et al., 1992; Lundemose et al., 1993; Mo et al., 1995; Suzuki et al., 2004; Leuzzi et al., 2005; Norville et al., 2011; Jana et al., 2012; Kim et al., 2014), as well as a MIP from the parasite *Trypanosoma cruzi* (Moro et al., 1995). PPIase activity of bacterial MIP proteins is commonly measured using the protease-coupled assay (Fischer et al., 1984; Fischer et al., 1992). Briefly, succinamide-Ala-Phe-Pro-Phe-p-nitroalanine peptide substrate is incubated with purified MIP (in the presence or absence of various inhibitor concentrations) for 4 min at 10°C in 35 mM HEPES/NaOH buffer (pH 7.8). The reaction is started by adding the isomer specific protease chymotrypsin, and the release of 4-nitroaniline is monitored by measuring absorbance at λ 390nm over 15 min.

MIP proteins offer targets for inhibitory drugs. PPIase activity can be inhibited by the fungal immunosuppressant macrocyclic compounds of rapamycin (sirolimus) and FK506 (tacrolimus) (Schreiber, 1991). Ng-MIP PPIase activity can be inhibited by rapamycin (Leuzzi et al., 2005). A recent study showed that pipecolic acid derivatives PipN3 and PipN4, originally shown to be inhibitors of Lp-MIP (Juli et al., 2011), inhibited the PPIase activity of Ng-MIP and affected the survival of gonococci in the presence of neutrophils (Reimer et al., 2016). PipN3 and PipN4 also inhibited the invasion and intracellular survival of meningococci in Detroit 562 epithelial cells. Subsequently, Seufert et al. established a library of pipecolic acid derivatives and used hot-spot analyses and exploratory docking experiments against Lp-Mip and Bp-Mip to suggest possible structure modifications to improve inhibitory activity (Seufert et al., 2016). Coupled with considerations of drug toxicity and stability, the authors synthesized and evaluated compounds in PPIase inhibition assays and identified compound S-5x as the most active inhibitor with the best safety profile. In general, such structure-activity analysis approaches, whereby the chemistry of potential inhibitors is modified to improve activity, can be applied to developing bacterial MIP PPIase inhibitors. Studies with purified MIP proteins *in vitro* would then select those drugs that can progress to *in vivo* activity and toxicity studies in animal infection models.

A proteomics study of the gonococcal response to sub-inhibitory concentrations of extended-spectrum cephalosporins such as ceftriaxone, found increased expression of Ng-MIP, and it was proposed that this up-regulated expression might be an adaptation or a tolerance mechanism of the bacteria in response to antibiotic stress (Nabu et al., 2017).

## Conclusions and Future Work

In summary, species of the genus *Neisseria* express a FKBP-type MIP protein in their OM. The key characteristics of MIP are that 1) anti-MIP antibodies that bind to the surfaces of pathogenic gonococci and meningococci are bactericidal, and 2) it may offer an OM-located drug target to help treat infections caused by antibiotic-resistant gonococci. Future work on MIP should focus on these two aspects. MIP deserves to be explored perhaps as a component of the first generation of modern antigen-specific gonococcal vaccines that are currently being developed (Wetzler et al., 2016). Perhaps revisiting the strategy to over-express truncated MIP in OMV should also be considered (Humbert and Christodoulides, 2018). What is unknown also is the peptide epitope sequence(s) that is/are responsible for inducing bactericidal antibodies and whether these sequences vary much between the different MIP proteins. Identifying these epitopes may offer the opportunity to use structural vaccinology approaches to develop refined subunit vaccines. Furthermore, even though studies suggest that MIP is a potential drug target, development of drugs to inhibit its virulence properties has not progressed. And finally, whether gonococci develop resistance to anti-MIP drugs is not known and should be addressed for any promising new therapeutics.

## Author Contributions

MC wrote the article and updated the literature from a previous in-depth review published elsewhere. 

## Conflict of Interest

The author declares that the article was written without any commercial or financial relationships that could be construed as potential conflicts of interest.

## Publisher’s Note

All claims expressed in this article are solely those of the authors and do not necessarily represent those of their affiliated organizations, or those of the publisher, the editors and the reviewers. Any product that may be evaluated in this article, or claim that may be made by its manufacturer, is not guaranteed or endorsed by the publisher.

## Supplementary Material

Supplementary Figure 1 Dendrogram for the 32 non-redundant Ng-MIP alleles.

Supplementary Figure 2 Dendrogram for the 149 non-redundant Nm-MIP alleles.

Supplementary Figure 3 Comparison of the structures of MIP from pathogenic and commensal *Neisseria* spp. Each structure used built on the template 1fd9.1. of *Legionella pneumophila* using SWISS-MODEL.

## Abbreviations

MIP, macrophage infectivity potentiator; FKBP, FK506 binding protein (FKBP) family of proteins; PPIase, peptidyl-prolyl cis/trans isomerase; PID, pelvic inflammatory disease; HIV, human immunodeficiency virus; SMD, systemic (invasive) meningococcal disease; Nm, *Neisseria meningitidis*; Ng*, Neisseria gonorrhoeae*; MLST, multi-locus sequence typing; Supplementary Material; BIGSbd, Bacterial Isolate Genome Sequence database; OM, outer membrane; OMV, outer membrane vesicle; Al(OH)_3_, aluminium hydroxide; Pip, pipecolic acid; Lp-MIP, *Legionella pneumophilia* MIP.URT, upper respiratory tract; CPS, capsule polysaccharide.
