# Supplementary figures and images for: Update on the Neisseria Macrophage Infectivity Potentiator-Like PPIase Protein
Source: Front Cell Infect Microbiol. 2022 Mar 22;12:861489. doi: 10.3389/fcimb.2022.861489 (PMC8981591; doi:10.3389/fcimb.2022.861489)

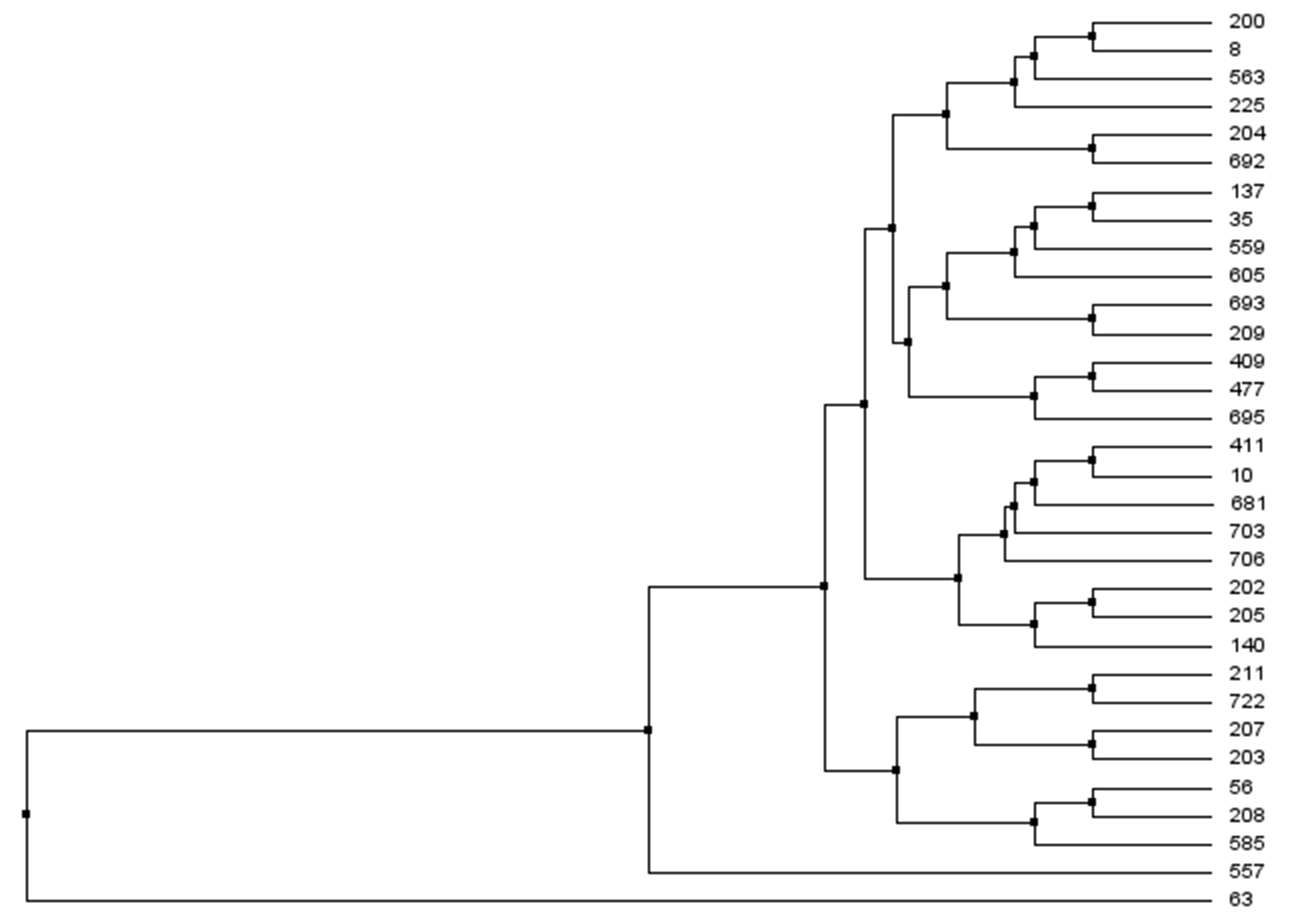

Supplement: Supplementary Figure 1 — Dendrogram for the 32 non-redundant Ng-MIP alleles. [file Image_1.tif]

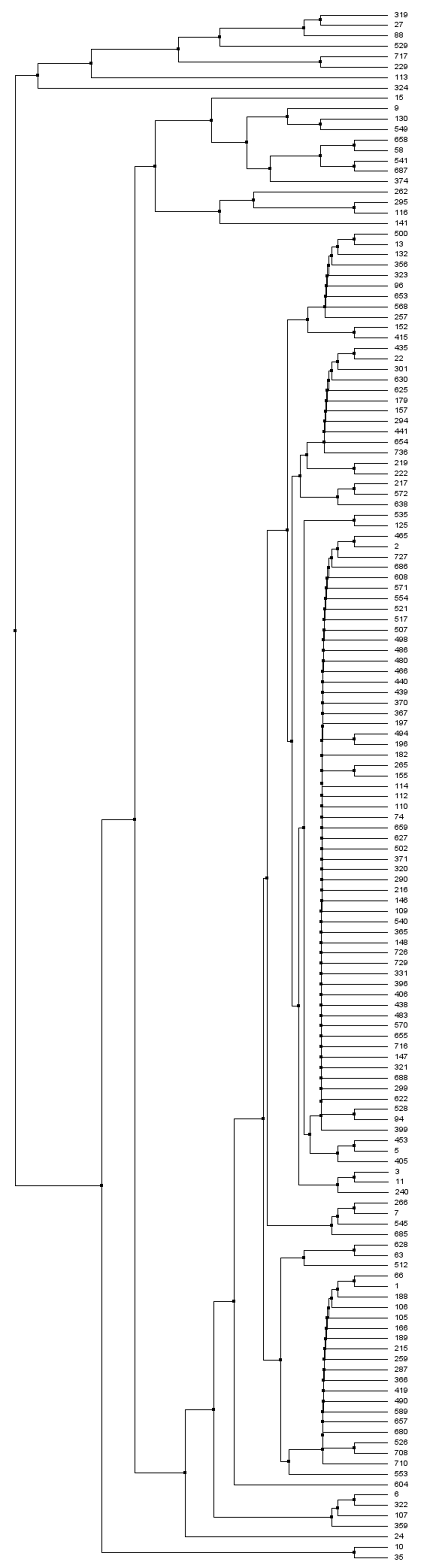

Supplement: Supplementary Figure 2 — Dendrogram for the 149 non-redundant Nm-MIP alleles. [file Image_2.tif]

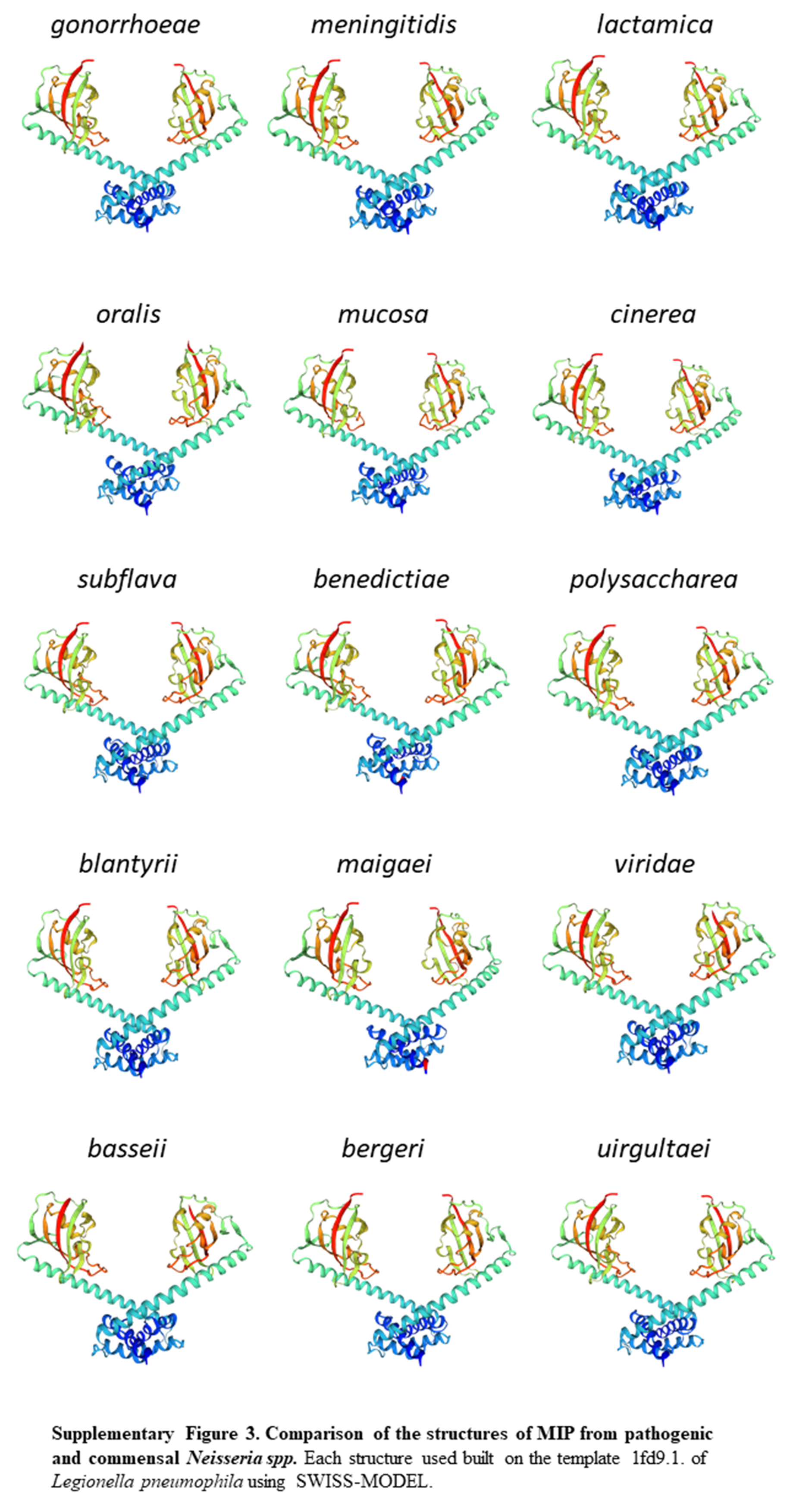

Supplement: Supplementary Figure 3 — Comparison of the structures of MIP from pathogenic and commensal Neisseria spp. Each structure used built on the template 1fd9.1. of Legionella pneumophila using SWISS-MODEL. [file Image_3.tif]
