# Supplementary material for: Update on the Neisseria Macrophage Infectivity Potentiator-Like PPIase Protein
Source: Front Cell Infect Microbiol. 2022 Mar 22;12:861489. doi: 10.3389/fcimb.2022.861489 (PMC8981591; doi:10.3389/fcimb.2022.861489)
Supplement: Supplementary file 5 [file Table_2.docx]

**Supplementary Dataset 2: a CLUSTAL multiple sequence alignment for Ng-MIP non-redundant alleles**

63 MNTIFKISALTLSAALALSACGKKEAAPASASEPAAASSAQGDTSSIGSTMQQASYAMGV 60

200 MNTIFKISALTLSAALALSACGKKEAAPASASEPS---AAQGDTSSIGSTMQQASYAMGV 57

557 MNTIFKISALTLSAALALSACGKKEAAPRICSEPAAASAAQGDTSSIGGTMQQASYAMGV 60

204 MNTIFKISALTLSAALALSACGKKEAAPASASEPAAASAAQGDTSSIGGTMQQASYAMGV 60

692 MNTIFKISALTLSAALALSACGKKEAAPASASEPAAASAAQGDTSSIGSTMQQASYAMGV 60

137 MNTIFKISALTLSAALALSACGKKEAAPASASEPAAASAAQGDTSSIGGTMQQASYAMGV 60

563 MNTIFKISALTLSAALALSACGKKEAAPASASEPAAASAAQGDTSSIGSTMQQASYAMGV 60

559 MNTIFKISALTLSAALALSACGKKEAVPASASEPAAASAAQGDTSSIGGTMQQASYAMGV 60

211 MNTIFKISALTLSAALALSACGKKEAAPAPASEPAAASAAQGDTSSIGSTMQQASYAMGV 60

722 MNTIFKISALTLSAALALSACGKKEAAPAPASEPAAASAAQGDTSSIGSTMQQASYAMGV 60

56 MNTIFKISALTLSAALALSACGKKEAAPAPASEPAAASAAQGDTFSIGGTMQQASYAMGV 60

585 MNTIFKISALTLSAALALSACGKKEAAPAPASEPAAASAAQGDTSSIGGTMQQASYAMGV 60

693 MNTIFKISALTLSAALALSACGKKEAAPASASEPAAASAAQGDTSSIGGTMQQASYAMGV 60

605 MNTIFKISALTLSAALALSACGKKEAAPASASEPAAASAAQGDTSSIGGTMQQASYAMGV 60

409 MNTIFKISALTLSAALALSACGKKEAAPASASEPAAASAAQGDTSSIGGTMQQASYAMGV 60

411 MNTIFKISALTLSAALALSACGKKEAAPASASEPAAASAAQGDTSSIGGTMQQASYAMGV 60

681 MNTIFKISALTLSAALALSACGKKEAAPASASEPAAASAAQGDTSSIGGTMQQASYAMGV 60

703 MNTIFKISALTLSAALALSACGKKEAAPASASEPAAASAAQGDTSSIGGTMQQASYAMGV 60

706 MNTIFKISALTLSAALALSACGKKEAAPASASEPAAASAAQGDTSSIGGTMQQASYAMGV 60

695 MNTIFKISALTLSAALALSACGKKEAAPASASEPAAASAAQGDTSSIGSTMQQASYAMGV 60

207 MNTIFKISALTLSAALALSACGKKEAVPAPASEPAAASAAQGDTSSIGSTMQQASYAMGV 60

225 MNTIFKISALTLSAALALSACGKKEAAPASASEPAAASAAQGDTSSIGSTMQQASYAMGV 60

477 MNTIFKISALTLSAALALSACGKKEAAPASASEPAAASAAQGDTSSIGGTMQQASYAMGV 60

209 MNTIFKISALTLSAALALSACGKKEAAPASASEPAAASAAQGDTSSIGGTMQQASYAMGV 60

208 MNTIFKISALTLSAALALSACGKKEAAPAPASEPAAASAAQGDTSSIGGTMQQASYAMGV 60

202 MNTIFKISALTLSAALALSACGKKEAAPAPASEPAAASAAQGDTSSIGGTMQQASYAMGV 60

203 MNTIFKISALTLSAALALSACGKKEAAPAPASEPAAASAAQGDTSSIGSTMQQASYAMGV 60

205 MNTIFKISALTLSAALALSACGKKEAAPAPASEPAAASAAQGDTSSIGSTMQQASYAMGV 60

8 MNTIFKISALTLSAALALSACGKKEAAPASASEPAAASAAQGDTSSIGSTMQQASYAMGV 60

140 MNTIFKISALTLSAALALSACGKKEAAPASASEPAAASAAQGDTSSIGSTMQQASYAMGV 60

10 MNTIFKISALTLSAALALSACGKKEAAPASASEPAAASAAQGDTSSIGGTMQQASYAMGV 60

35 MNTIFKISALTLSAALALSACGKKEAAPASASEPAAASAAQGDTSSIGGTMQQASYAMGV 60

**************************.* .***: :***** ***.***********

63 DIGRSLKQMKEQGAEIDLKVFTEAMQAVYDGKEIKMTEEQAQEVMMKFLQEQQAKAVEKH 120

200 DIGRSLKQMKEQGAEIDLKVFTDAMQAVYDGKEIKMTEEQAQEVMMKFLQEQQAKAVEKH 117

557 DIGRSLKQMKEQGAEIDLKVFTDAMQAVYDGKEIKMTEEQAQEVMMKFLQEQQAKAVEKH 120

204 DIGRSLKQMKEQGAEIDLKVFTDAMQAVYDGKEIKMTEEQAQEVMMKFLQEQQAKAVEKH 120

692 DIGRSLKQMKEQGAEIDLKVFTDAMQAVYDGKEIKMTEEQAQEVMMKFLQEQQAKAVEKH 120

137 DIGRSLKQMKEQGAEIDLKVFTDAMQAVYDGKEIKMTEEQAQEVMMKFLQEQQAKAVEKH 120

563 DIGRSLKQMKEQGAEIDLKVFTDAMQAVYDGKEIKMTEEQAQEVMMKFLQEQQAKAVEKH 120

559 DIGRSLKQMKEQGAEIDLKVFTDAMQAVYDGKEIKMTEEQAQEVMMKFLQEQQAKAVEKH 120

211 DIGRSLKQMKEQGAEIDLKVFTDAMQAVYDGKEIKMTEEQVQEVMMKFLQEQQAKAVEKH 120

722 DIGRSLKQMKEQGAEIDLKVFTDAMQAVYDGKEIKMTEEQVQEVMMKFLQEQQAKAVEKH 120

56 DIGRSLKQMKEQGAEIDLKVFTDAMQAVYDGKEIKMTEEQAQEVMMKFLQEQQAKAVEKH 120

585 DIGRSLKQMKEQGAEIDLKVFTDAMQAVYDGKEIKMTEEQAQEVMMKFLQEQQAKAVEKH 120

693 DIGRSLKQMKEQGAEIDLKVFTDAMQAVYDGKEIKMTEEQVQEVMMKFLQEQQAKAVEKH 120

605 DIGRSLKQMKEQGAEIDLKVFTDAMQAVYDGKEIKMTEEQAQEVMMKFLQEQQAKAVEKH 120

409 DIGRSLKQMKEQGAEIDLKVFTDAMQAVYDGKEIKMTEEQAQEVMMKFLQEQQAKAVEKH 120

411 DIGRSLKQMKEQGAEIDLKVFTDAMQAVYDGKEIKMTEEQAQEVMMKFLQEQQAKAVEKH 120

681 DIGRSLKQMKEQGAEIDLKVFTDAMQAVYDGKEIKMTEEQAQEVMMKFLQEQQAKAVEKH 120

703 DIGRSLKQMKEQGAEIDLKVFTDAMQAVYDGKEIKMTEEQAQEVMMKFLQEQQAKAVEKH 120

706 DIGRSLKQMKEQGAEIDLKVFTDAMQAVYDGKEIKMTEEQAQEVMMKFLQEQQAKAVEKH 120

695 DIGRSLKQMKEQGAEIDLKVFTDAMQAVYDGKEIKMTEEQAQEVMMKFLQEQQAKAVEKH 120

207 DIGRSLKQMKEQGAEIDLKVFTDAMQAVYDGKEIKMTEEQAQEVMMKFLQEQQAKAVEKH 120

225 DIGRSLKQMKEQGAEIDLKVFTDAMQAVYDGKEIKMTEEQAQEVMMKFLQEQQAKAVEKH 120

477 DIGRSLKQMKEQGAEIDLKVFTDAMQAVYDGKEIKMTEEQAQEVMMKFLQEQQAKAVEKH 120

209 DIGRSLKQMKEQGAEIDLKVFTDAMQAVYDGKEIKMTEEQVQEVMMKFLQEQQAKAVEKH 120

208 DIGRSLKQMKEQGAEIDLKVFTDAMQAVYDGKEIKMTEEQAQEVMMKFLQEQQAKAVEKH 120

202 DIGRSLKQMKEQGAEIDLKVFTDAMQAVYDGKEIKMTEEQAQEVMMKFLQEQQAKAVEKH 120

203 DIGRSLKQMKEQGAEIDLKVFTDAMQAVYDGKEIKMTEEQAQEVMMKFLQEQQAKAVEKH 120

205 DIGRSLKQMKEQGAEIDLKVFTDAMQAVYDGKEIKMTEEQAQEVMMKFLQEQQAKAVEKH 120

8 DIGRSLKQMKEQGAEIDLKVFTDAMQAVYDGKEIKMTEEQAQEVMMKFLQEQQAKAVEKH 120

140 DIGRSLKQMKEQGAEIDLKVFTDAMQAVYDGKEIKMTEEQAQEVMMKFLQEQQAKAVEKH 120

10 DIGRSLKQMKEQGAEIDLKVFTDAMQAVYDGKEIKMTEEQAQEVMMKFLQEQQAKAVEKH 120

35 DIGRSLKQMKEQGAEIDLKVFTDAMQAVYDGKEIKMTEEQAQEVMMKFLQEQQAKAVEKH 120

**********************:*****************.*******************

63 KADAKANKEKGEAFLKENAAKDGVKTTASGLQYKITKQGEGKQPSKDDIVTVEYEGRLID 180

200 KADAKANKEKGEAFLKENAAKDGVKTTASGLQYKITKQGEGKQPTKDDIVTVEYEGRLID 177

557 KADAKANKEKGEAFLKENAAKDGVKTTASGLQYKITKQGKGKQPTKDDIVTVEYEGRLID 180

204 KADAKANKEKGEAFLKENAAKDGVKTTASGLQYKITKQGEGKQPTKDDIVTVEYEGRLID 180

692 KADAKANKEKGEAFLKENAAKDGVKTTASGLQYKITKQGEGKQPTKDDIVTVEYEGRLID 180

137 KADAKANKEKGEAFLKENAAKDGVKTTASGLQYKITKQGEGKQPTKDDIVTVEYEGRLID 180

563 KADAKANKEKGEAFLKENAAKDGVKTTASGLQYKITKQGEGKQPTKDDIVTVEYEGRLID 180

559 KADAKANKEKGEAFLKENAAKDGVKTTASGLQYKITKQGEGKQPTKDDIVTVEYEGRLID 180

211 KADAKANKEKGEAFLKENAAKDGVKTTASGLQYKITKQGKGKQPTKDDIVTVEYEGRLID 180

722 KADAKANKEKGEAFLKENAAKDGVKTTASGLQYKITKQGEGKQPTKDDIVTVEYEGRLID 180

56 KADAKANKEKGEAFLKENAAKDGVKTTASGLQYKITKQGEGKQPTKDDIVTVEYEGRLID 180

585 KADAKANKEKGEAFLKENAAKDGVKTTASGLQYKITKQGEGKQPTKDDIVTVEYEGRLID 180

693 KADAKANKEKGEAFLKENAAKDGVKTTASGLQYKITKQGKGKQPTKDDIVTVEYEGRLID 180

605 KADAKANKEKGEAFLKENVAKDGVKTTASGLQYKITKQGEGKQPTKDDIVTVEYEGRLID 180

409 KADAKANKEKGEAFLKENAAKDGVKTTASGLQYKITKQGKGKQPTKDDIVTVEYEGRLID 180

411 KADAKANKEKGEAFLKENAAKDGVKTTASGLQYKITKQGKGKQPTKDDIVTVEYEGRLID 180

681 KADAKANKEKGEAFLKENAAKDGVKTTASGLQYKITKQGKGKQPTKDDIVTVEYEGRLID 180

703 KADAKTNKEKGEAFLKENAAKDGVKTTASGLQYKITKQGKGKQPTKDDIVTVEYEGRLID 180

706 KADAKANKEKGEAFLKENAAKDGVKTTASGLQYKITKQGKGKQPTKDDIVTVEYEGRLID 180

695 KADAKANKEKGEAFLKENAAKDGVKTTASGLQYKITKQGEGKQPTKDDIVTVEYEGRLID 180

207 KADAKANKEKGEAFLKENAAKDGVKTTASGLQYKITKQGEGKQPTKDDIVTVEYEGRLID 180

225 KADAKANKEKGEAFLKENAAKDGVKTTASGLQYKITKQGEGKQPTKDDIVTVEYEGRLID 180

477 KADAKANKEKGEAFLKENAAKDGVKTTASGLQYKITKQGEGKQPTKDDIVTVEYEGRLID 180

209 KADAKANKEKGEAFLKENAAKDGVKTTASGLQYKITKQGEGKQPTKDDIVTVEYEGRLID 180

208 KADAKANKEKGEAFLKENAAKDGVKTTASGLQYKITKQGEGKQPTKDDIVTVEYEGRLID 180

202 KADAKANKEKGEAFLKENAAKDGVKTTASGLQYKITKQGKGKQPTKDDIVTVEYEGRLID 180

203 KADAKANKEKGEAFLKENAAKDGVKTTASGLQYKITKQGEGKQPTKDDIVTVEYEGRLID 180

205 KADAKANKEKGEAFLKENAAKDGVKTTASGLQYKITKQGKGKQPTKDDIVTVEYEGRLID 180

8 KADAKANKEKGEAFLKENAAKDGVKTTASGLQYKITKQGEGKQPTKDDIVTVEYEGRLID 180

140 KADAKANKEKGEAFLKENAAKDGVKTTASGLQYKITKQGKGKQPTKDDIVTVEYEGRLID 180

10 KADAKANKEKGEAFLKENAAKDGVKTTASGLQYKITKQGKGKQPTKDDIVTVEYEGRLID 180

35 KADAKANKEKGEAFLKENAAKDGVKTTASGLQYKITKQGEGKQPTKDDIVTVEYEGRLID 180

*****:************.********************:****:***************

63 GTVFDSSKANGGPVTFPLSQVIPGWTEGVQLLKEGGEATFYIPPNLAYREQGAGDKIGPN 240

200 GTVFDSSKANGGPATFPLSQVIPGWTEGVRLLKEGGEATFYIPSNLAYREQGAGEKIGPN 237

557 GTVFDSSKANGGPATFPLSQVIPGWTEGVRLLKEGGEATFYIPSNLAYREQGAGEKIGPN 240

204 GTVFDSSKANGGPATFPLNQVIPGWTEGVRLLKEGGEATFYIPSNLAYREQGAGEKIGPN 240

692 GTVFDSSKANGGPATFPLNQVIPGWTEGVRLLKEGGEATFYIPSNLAYREQGAGEKIGPN 240

137 GTVFDSSKANGGPAPFPLSQVIPGWTEGVRLLKEGGEATFYIPSNLAYREQGAGEKIGPN 240

563 GTVFDSSKANGGPATFPLSQVIPGWTEGVRLLKEGGEATFYIPSNLAYREQGAGEKIGPN 240

559 GTVFDSSKANGGPATFPLSQVIPGWTEGVRLLKEGGEATFYIPSNLAYREQGAGEKIGPN 240

211 GTVFDSSKANGGPATFPLSQVIPGWTEGVRLLKEGGEATFYIPSNLAYREQGAGEKIGPN 240

722 GTVFDSSKANGGPATFPLSQVIPGWTEGVRLLKEGGEATFYIPSNLAYREQGAGEKIGPN 240

56 GTVFDSSKANGGPATFPLSQVIPGWTEGVRLLKEGGEATFYIPSNLAYREQGAGEKIGPN 240

585 GTVFDSSKANGGPATFPLSQVIPGWTEGVRLLKEGGEATFYIPSNLAYREQGAGEKIGPN 240

693 GTVFDSSKANGGPATFPLSQVIPGWTEGVRLLKEGGEATFYIPSNLAYREQGAGEKIGPN 240

605 GTVFDSSKANGGPATFPLSQVIPGWTEGVRLLKEGGEATFYIPSNLAYREQGAGEKIGPN 240

409 GIVFDSSKANGGPATFPLSQVIPGWTEGVRLLKEGGEATFYIPSNLAYREQGAGEKIGPN 240

411 GTVFDSSKANGGPATFPLSQVIPGWTEGVRLLKEGGEATFYIPSNLAYREQGAGEKIGPN 240

681 GTVFDSSKANGGPATFPLSQVIPGWTEGVRLLKEGGEATFYIPSNLAYREQGAGEKIGPN 240

703 GTVFDSSKANGGPATFPLSQVIPGWTEGVRLLKEGGEATFYIPSNLAYREQGAGEKIGPN 240

706 GTVFDSSKANGGLATFPLSQVIPGWTEGVRLLKEGGEATFYIPSNLAYREQGAGEKIGPN 240

695 GIVFDSSKANGGPATFPLSQVIPGWTEGVRLLKEGGEATFYIPSNLAYREQGAGEKIGPN 240

207 GTVFDSSKANGGPATFPLSQVIPGWTEGVRLLKEGGEATFYIPSNLAYREQGAGEKIGPN 240

225 GTVFDSSKANGGPVTFPLSQVIPGWTEGVRLLKEGGEATFYIPSNLAYREQGAGEKIGPN 240

477 GIVFDSSKANGGPATFPLSQVIPGWTEGVRLLKEGGEATFYIPSNLAYREQGAGEKIGPN 240

209 GTVFDSSKANGGPATFPLSQVIPGWTEGVRLLKEGGEATFYIPSNLAYREQGAGEKIGPN 240

208 GTVFDSSKANGGPATFPLSQVIPGWTEGVRLLKEGGEATFYIPSNLAYREQGAGEKIGPN 240

202 GTVFDSSKANGGPATFPLSQVIPGWTEGVRLLKEGGEATFYIPSNLAYREQGAGEKIGPN 240

203 GTVFDSSKANGGPATFPLSQVIPGWTEGVRLLKEGGEATFYIPSNLAYREQGAGEKIGPN 240

205 GTVFDSSKANGGPATFPLSQVIPGWTEGVRLLKEGGEATFYIPSNLAYREQGAGEKIGPN 240

8 GTVFDSSKANGGPATFPLSQVIPGWTEGVRLLKEGGEATFYIPSNLAYREQGAGEKIGPN 240

140 GTVFDSSKANGGPATFPLSQVIPGWTEGVRLLKEGGEATFYIPSNLAYREQGAGEKIGPN 240

10 GTVFDSSKANGGPATFPLSQVIPGWTEGVRLLKEGGEATFYIPSNLAYREQGAGEKIGPN 240

35 GTVFDSSKANGGPATFPLSQVIPGWTEGVRLLKEGGEATFYIPSNLAYREQGAGEKIGPN 240

* ********** . ***.**********:************* **********:*****

63 ATLVFDVKLVKIGAPENAPAKQPAQVDIKKVN 272

200 ATLVFDVKLVKIGAPENAPAKQPDQVDIKKVN 269

557 ATLVFDVKLVKIGAPENAPAKQPDQVDIKKVN 272

204 ATLVFDVKLVKIGAPENAPAKQPDQVDIKKVN 272

692 ATLVFDVKLVKIGAPENAPAKQPDQVDIKKVN 272

137 ATLVFDVKLVKIGAPENAPAKQPDQVDIKKVN 272

563 ATLVFDVKLVKIGAPENVPAKQPDQVDIKKVN 272

559 ATLVFDVKLVKIGAPENAPAKQPDQVDIKKVN 272

211 ATLVFDVKLVKIGAPENAPAKQPDQVDIKKVN 272

722 ATLVFDVKLVKIGAPENAPAKQPDQVDIKKVN 272

56 ATLVFDVKLVKIGAPENAPAKQPDQVDIKKVN 272

585 ATLVFDVKLVKIGAPENAPAKQLDQVDIKKVN 272

693 ATLVFDVKLVKIGAPENAPAKQPDQVDIKKVN 272

605 ATLVFDVKLVKIGAPENAPAKQPDQVDIKKVN 272

409 ATLVFDVKLVKIGAPENAPAKQPDQVDIKKVN 272

411 ATLVFDVKLVKIGAPENAPAKQLDQVDIKKVN 272

681 ATLVFDVKLVKIGAPENAPAKQPDQVDIKQVN 272

703 ATLVFDVKLVKIGAPENAPAKQPDQVDIKKVN 272

706 ATLVFDVKLVKIGAPENAPAKQPDQVDIKKVN 272

695 ATLVFDVKLVKIGAPENAPAKQPDQVDIKKVN 272

207 ATLVFDVKLVKIGAPENAPAKQPDQVDIKKVN 272

225 ATLVFDVKLVKIGAPENAPAKQPDQVDIKKVN 272

477 ATLVFDVKLVKIGAPENAPAKQPDQVDIKKVN 272

209 ATLVFDVKLVKIGAPENAPAKQPDQVDIKKVN 272

208 ATLVFDVKLVKIGAPENAPAKQPDQVDIKKVN 272

202 ATLVFDVKLVKIGAPENAPAKQPDQVDIKKVN 272

203 ATLVFDVKLVKIGAPENAPAKQPDQVDIKKVN 272

205 ATLVFDVKLVKIGAPENAPAKQPDQVDIKKVN 272

8 ATLVFDVKLVKIGAPENAPAKQPDQVDIKKVN 272

140 ATLVFDVKLVKIGAPENAPAKQPDQVDIKKVN 272

10 ATLVFDVKLVKIGAPENAPAKQPDQVDIKKVN 272

35 ATLVFDVKLVKIGAPENAPAKQPDQVDIKKVN 272

*****************.**** *****:**
