# Supplementary material for: Update on the Neisseria Macrophage Infectivity Potentiator-Like PPIase Protein
Source: Front Cell Infect Microbiol. 2022 Mar 22;12:861489. doi: 10.3389/fcimb.2022.861489 (PMC8981591; doi:10.3389/fcimb.2022.861489)
Supplement: Supplementary file 7 [file Table_4.docx]

**Supplementary Dataset 4. A CLUSTAL alignment of non-redundant Nm-MIP sequences**

15 MNTIFKISALTLSAALALSACGKKEAAPA-ATASEPAAASAAQGDTSSIGSTMQQASYAM 59

319 MNTIFKISALTLSAALALSACGKKEAA------SEPAAASAAQGDTSSIGSTMQQASYAM 54

27 MNTIFKISALTLSAALALSACGKKEAASASASASEPAAASAAQGDTSSIGSTMQQASYAM 60

88 MNTIFKISALTLSAALALSACGKKEAAPA--SASEPAAASAAQGDTSSIGSTMQQASYAM 58

113 MNKIFKISALTLAATLALSACGKKENVPAS-SASEPAAASAAQGDTSSIGNTMQQASYAM 59

717 MNKIFKISALTLAATLALSACGKKEAAPA--SASEPAAASAAQGDTSSIGSTMQQASYAM 58

229 MNKIFKISALTLAATLALSACGKKEAAPA--SASEPAAASAAQGDTSSIGSTMQQASYAM 58

324 MNTIFKISALTLAAALALSACGKKEAAPAA-SASEPAAASAAQGDTSSIGSTMQQASYAM 59

262 MNTIFKISALTLSATLALSACGKKEAAP-A-SASEPAAASAAQGDTSSIGSTMQQASYAM 58

9 MNTIFKISALTLSAALALSACGKKEAASAA-TASEPAAASAAQGDTSSIGSTMQQASYAM 59

295 MNTIFKISALTLSAALALSACGKKEAA------SEPAAASAAQGDTSSIGSTMQQASYAM 54

130 MNTIFKISALTLSAALALSACGKKEAA------SEPAAASAAQGDTSSIGSTMQQASYAM 54

658 MNTIFKISALTLSAALALSACGKKEAAPA-ATASEPAAASAAQGDTSSIGSTMQQASYAM 59

58 MNTIFKISALTLSAALALSACGKKEAAPA-ATASEPAAASAAQGDTSSIGSTMQQASYAM 59

374 MNTIFKISALTLSAALALSACGKKEAAPA-S-ASEPAAASSAQGDTSSIGSTMQQASYAM 58

500 MNTIFKISALTLSAALALSACGKKEAAPA-S-ASEPAAASSAQGDTSSIGSTMQQASYAM 58

10 MNTIFKISALTLSAALALSACGKKEAAPA-S-ASEPAAASAAQGDTSSIGGTMQQASYAM 58

35 MNTIFKISALTLSAALALSACGKKEAAPA-S-ASEPAAASAAQGDTSSIGGTMQQASYAM 58

141 MNTIFKISALTLSAALALSACGKKEAAPA-S-ASEPAAASSAQGDTSSIGSTMQQASYAM 58

628 MNTIFKISALTLSAALALSACGKKEAAPA-S-ASEPAAASSAQGDTSSIGSTMQQASYAM 58

435 MNTIFKISALTLSAALALSACGKKEAAPA-S-ASEPAAASAAQGDTSSIGSTMQQASYAM 58

132 MNTIFKISALTLSAALALSACGKKEAAPA-S-ASEPAAASAAQGDTSSIGSTMQQASYAM 58

356 MNTIFKISALTLSAALALSACGKKEAAPA-S-ASEPAAASSAQGDTSSIGSTMQQASYAM 58

6 MNTIFKISALTLSAALALSACGKKEAAPAS--ASEPAAASSAQGDTSSIGSTMQQASYAM 58

322 MNTIFKISALTLFAALALSACGKKEAAPAS--ASEPAAASSAQGDTSSIGSTMQQASYAM 58

301 MNTIFKISALTLSAALALSACGKKEAAPAS--ASEPAAASAAQGDTSSIGSTMQQASYAM 58

630 MNTIFKISALTLSAALALSACGKKEAAPAS--ASEPAAASAAQGETSSIGSTMQQASYAM 58

625 MNTIFKISALTLSAALALSACGKKEAAPAS--ASEPATASAAQGDTSSIGSTMQQASYAM 58

179 MNTIFKISALTLSAALALSACGKKEAAPAS--ASEPAAASAAQGDTSSIGSTMQQASYAM 58

63 MNTIFKISALTLSAALALSACGKKEAAPAS--ASEPAAASSAQGDTSSIGSTMQQASYAM 58

535 MNTIFKISALTLSAALALSACGKKEAAPAS--ASEPAAASSAQGDTSSIGSTMQQASYAM 58

125 MNTIFKISALTLSAALALSACGKKEAAPAS--ASEPAAASSAQGDTSSIGSTMQQASYAM 58

217 MNTIFKISALTLSAALALSACGKKEAAPAS--ASEPAAASAAQGDTSSIGSTMQQASYAM 58

465 MNTIFKISALTLSAALALSACGKKEAAPAS--ASEPAAASSAQGDTSSIGSTMQQASYAM 58

66 MNTIFKISALTLSAALALSACGKKEAAPAS--ASEPAAASSAQGDTSSIGSTMQQASYAM 58

3 MNTIFKISALTLSAALALSACGKKEAAPAS--ASEPAAASSAQGDTSSIGSTMQQASYAM 58

11 MNTIFKISALTLSAALALSACGKKEAAPAS--ASEPAAASSAQGDTSSIGSTMQQASYAM 58

453 MNTIFKISALTLSAALALSACGKKEAASAS--ASEPAAASSAQDDTSSIGSTMQQASYAM 58

5 MNTIFKISALTLSAALALSACGKKEAAPAS--ASEPAAASSAQDDTSSIGSTMQQASYAM 58

727 MNTIFKISALTLSAALALSACGKKEAAPAS--ASEPAAASSAQGDTSSIGSTMQQASYAM 58

686 MNTIFKISALTLSAALALSACGKKEAAPAS--ASEPAAASSAQGDTSSIGSTMQQASYAM 58

608 MNTIFKISALTLSAALALSACGKKEAAPAS--ASEPAAASSAQGDTSSIGSTMQQASYAM 58

571 MNTIFKISALTLSAALALSACGKKEAAPAS--ASEPAAASSAQGDTSSIGSTMQQASYAM 58

554 MNTIFKISALTLSAALALSACGKKEAAPAS--ASEPAAASSAQGDTSSIGSTMQQASYAM 58

521 MNTIFKISALTLSAALALSACGKKEAAPAS--ASEPAAASSAQGDTSSIGSTMQQASYAM 58

517 MNTIFKISALTLSAALALSACGKKEAAPAS--ASEPAAASSAQGDTSSIGSTMQQASYAM 58

507 MNTIFKISALTLSAALALSACGKKEAAPAS--ASEPAAASSAQGDTSSIGSTMQQASYAM 58

498 MNTIFKISALTLSAALALSACGKKEAAPAS--ASEPAAASSAQGDTSSIGSTMQQASYAM 58

486 MNTIFKISALTLSAALALSACGKKEAAPAS--ASEPAAASSAQGDTSSIGSTMQQASYAM 58

480 MNTIFKISALTLSAALALSACGKKEAAPAS--ASEPAAASSAQGDTSSIGSTMQQASYAM 58

466 MNTIFKISALTLSAALALSACGKKEAAPAS--ASEPAAASSAQGDTSSIGSTMQQASYAM 58

440 MNTIFKISALTLSAALALSACGKKEAAPAS--ASEPAAASSAQGDTSSIGSTMQQASYAM 58

439 MNTIFKISALTLSAALALSACGKKEAAPAS--ASEPAAASSAQGDTSSIGSTMQQASYAM 58

370 MNTIFKISALTLSVALALSACGKKEAAPAS--ASEPAAASSAQGDTSSIGSTMQQASYAM 58

367 MNTIFKISALTLSAALALSACGKKEAAPAS--ASEPAAASSAQGDTSSIGSTMQQASYAM 58

219 MNTIFKISALTLSAALALSACGKKEAAPAS--ASEPAAASSAQGDTSSIGSTMQQASYAM 58

197 MNTIFKISALTLSAALALSACGKKEAAPAS--ASEPAAASSAQGDTSSIGSTMQQASYAM 58

494 MNTIFKISALTLSAALALSACGKKEAAPAS--ASEPAAASSAQGDTSSIGSTMQQASYAM 58

196 MNTIFKISALTLSAALALSACGKKEAAPAS--ASEPAAASSAQGDTSSIGSTMQQASYAM 58

182 MNTIFKISALTLSAALALSACGKKEAAPAS--ASEPAAASSAQGDTSSIGSTMQQASYAM 58

265 MNTIFKISALTLSAALALSACGKKEAAPAS--ASEPAAASSAQGDTSSIGSTMQQASYAM 58

155 MNTIFKISALTLSAALALSACGKKEAAPAS--ASEPAAASSAQGDTSSIGSTMQQASYAM 58

114 MNTIFKISALTLSAALALSACGKKEAAPAS--ASEPAAASSAQGDTSSIGSTMQQASYAM 58

112 MNTIFKISALTLSAALALSACGKKEAAPAS--ASEPAAASSAQGDTSSIGSTMQQASYAM 58

110 MNTIFKISALTLSAALALSACGKKEAAPAS--ASEPAAASSAQGDTSSIGSTMQQASYAM 58

74 MNTIFKISALTLSAALALSACGKKEAAPAS--ASEPAAASSAQGDTSSIGSTMQQASYAM 58

659 MNTIFKISALTLSAALALSACGKKEAAPAS--ASEPAAASSAQGDTSSIGSTMQQASYAM 58

627 MNTIFKISALTLSAALALSACGKKEAAPAS--ASEPAAASSAQGDTSSIGSTMQQASYAM 58

502 MNTIFKISALTLSAALALSACGKKEAAPAS--ASEPAAASSAQGDTSSIGSTMQQASYAM 58

371 MNTIFKISALTLSAALALSACGKKEAAPAS--ASEPAAASSAQGDTSSIGSTMQQASYAM 58

320 MNTIFKISALTLSAALAFSACGKKEAAPAS--ASEPAAASSAQGDTSSIGSTMQQASYAM 58

290 MNTIFKISALTLSAALALSACGKKEAAPAS--ASEPAAASSAQGDTSSIGSTMQQASYAM 58

216 MNTIFKISALTLSAALALSACGKKEAAPAS--ASEPAAASSAQGDTSSIGSTMQQASYAM 58

146 MNTIFKISALTLSAALALSACGKKEAAPAS--ASEPAAASSAQGDTSSIGSTMQQASYAM 58

109 MNTIFKISALTLSAALALSACGKKEATPAS--ASEPAAASSAQGDTSSIGSTMQQASYAM 58

572 MNTIFKISALTLSAALALSACGKKEAAPAS--ASEPAAASSAQGDTSSIGSTMQQASYAM 58

540 MNAIFKISALTLSAALALSACGKKEAAPAS--ASEPAAASSAQGDTSSIGSTMQQASYAM 58

365 MNTIFKISALTLSAALALSACGKKEAAPAS--ASEPAAASSAQGDTSSIGSTMQQASYAM 58

148 MNTIFKISALTLSAALALSACGKKEAAPAS--ASEPAAASSAQGDTSSIGSTMQQASYAM 58

726 MNTIFKISALTLSAALALSACGKKEAAPAS--ASEPAAASSAQGDTSSIGSTMQQASYAM 58

729 MNTIFKISALTLSAALALSACGKKEAAPAS--ASEPAAASSAQGDTSSIGSTMQQASYAM 58

331 MNTIFKISALTLSAALALSACGKKEAAPAS--ASEPAAASSAQGDTSSIGSTMQQASYAM 58

396 MNTIFKISALTLSAALALSACGKKEAAPAS--ASEPAAASSEQGDTSSIGSTMQQASYAM 58

406 MNTIFKISALTLSAALALSACGKKEAAPAS--ASEPAAASSAQGDTSSIGSTMQQASYAM 58

438 MNTIFKISALTLSATLALSACGKKEAAPAS--ASEPAAASSAQGDTSSIGSTMQQASYAM 58

483 MNTIFKISALTLSAALALSACGKKEAAPAS--ASEPAAASSAQGDTSSIGSTMQQASYAM 58

570 MNTIFKISALTLSAALALSACGKKEAAPAS--ASEPAAASSAQGDTSSIGSTMQQASYAM 58

655 MNTIFKISALTLSAALALSACGKKEAAPAS--ASKPAAASSAQGDTSSIGSTMQQASYAM 58

716 MNTIFKISALTLSAALALSACGKKEAAPAS--ASEPAAASSAQGDTSSIGSAMQQASYAM 58

147 MNTIFKISALTLSAALALSACGKKEAAPAS--ASEPAAASSAQGDTSSIGSTMQQASYAM 58

321 MNTIFKISALTLSAALALSACGKKEAAPAS--ASEPAAASSAQGDTSSIGGTMQQASYAM 58

688 MNTIFKISALTLSAALALSACGKKEAAPAS--ASEPAAASSAQGDTSSIGSTMQQASYAM 58

299 MNTIFKISALTLSAALALSACGKKEAAPAS--ASEPAAASSAQGDTSSIGSTMQQASYAM 58

622 MNTIFKISALTLSAALALSACGKKEAAPAF--ASEPAAASSAQGDTSSIGSTMQQASYAM 58

405 MNTIFKISALTLSAALALSACGKKEAASAS--ASEPAAASSAQGDTSSIGSTMQQASYAM 58

528 MNTIFKISALTLSAALALSACGKKEAAPAS--VSEPAAASSAQGDTSSIGSTMQQASYAM 58

94 MNTIFKISALTLSAALALSACGKKEAAPAS--TSEPAAASSAQGDTSSIGSTMQQASYAM 58

399 MNTIFKISALTLSAALALSACGKKEAAPAS--AFEPAAASSAQGDTSSIGSTMQQASYAM 58

2 MNTIFKISALTLSAALALSACGKKEAAPAS--ASEPAAASSAQGDTSSIGSTMQQASYAM 58

152 MNTIFKISALTLSAALVLSACGKKEAAPAS--ASEPAAASSAQGDTSSIGSTMQQASYAM 58

415 MNTIFKISALTLSAALVLSACGKKEAAPAS--ASEPAAASSAQGDTSSIGSTMQQASYAM 58

323 MNTIFKISALTLSAALALSACGKKEAAPAS--ASEPAAASSAQGDTSSIGSTMQQASYAM 58

96 MNTIFKISALTLSAALALSACGKKEAAPAS--ASEPAAASSAQGDTSSIGSTMQQASYAM 58

529 MNTIFKISALTLSAALALSACGKKEAAPAS--ASEPAAASSAQGDTSSIGSTMQQASYAM 58

541 MNTIFKISALTLSAALALSACGKKEAAPAS--ASEPAAASSAQGDTSSIGSTMQQASYAM 58

687 MNTIFKISALTLSAALALSACGKKEAAPAS--ASEPAAASSAQGDTSSIGSTMQQASYAM 58

107 MNTIFKISALTLSAALALSACGKKEAA------SEPAAASAAQGDTSSIGSTMQQASYAM 54

266 MNTIFKISALTLSAALALSACGKKEAA------SEPAAASAAQGDTSSIGSTMQQASYAM 54

545 MNTIFKISALTLSAALALSACGKKEAA------SEPAAASAAQGDTSSIGSTMQQASYAM 54

685 MNTIFKISALTLSAALALSACGKKEAA------SEPAAASAAQGDTSSIGSTMQQASYAM 54

7 MNTIFKISALTLSAALALSACGKKEAA------SEPAAASAAQGDTSSIGSTMQQASYAM 54

549 MNTIFKISALTLSAALALSACGKKEAA------SEPAAASAAQGDTSSIGSTMQQASYAM 54

553 MNTIFKISALTLSAALALSACGKKEAA------SEPAAASAAQGDTSSIGSTMQQASYAM 54

222 MNTIFKISALTLSAALALSACGKKEAA------SEPAAASAAQGDTSSIGSTMQQASYAM 54

157 MNTIFKISALTLSAALALSACGKKEAA------SEPAAASAAQGDTSSIGSMMQQASYAM 54

294 MNTIFKISALTLSAALALSACGKKEAA------SEPAAASAAQGDTSSIGSTMQQASYAM 54

441 MNTIFKISALTLSAALALSACGKKEAA------SEPAAASAAQGDTSSIGSTMQQASYAM 54

654 MNTIFKISALTLSAALALSACGKKEAA------SEPAAASAAQGDTSSIGSTMQQASYAM 54

736 MNTIFKISALTLSAALALSACGKKEAA------SEPAAASAAQGDTSSIGSTMQQASYAM 54

240 MNTIFKISALTLSAALALSACGKKEAA------SEPAAASAAQGDTSSIGSTMQQASYAM 54

22 MNTIFKISALTLSAALALSACGKKEAA------SEPAAASAAQGDTSSIGSTMQQASYAM 54

188 MNTIFKISALTLSAALALSACGKKEAA------SEPAAASAAQGDTSSIGSTMQQASYAM 54

638 MNTIFKISALTLSAALALSACGKKEAA------SEPAAASAAQGDTSSIGSTMQQASYAM 54

106 MNTIFKISALTLSAALALSACGKKEAA------SEPAAASAAQGDTSSIGSTMQQASYAM 54

105 MNTIFKISALTLSAALALSACGKKEAA------SEPAAASAAQGDTSSIGSTMQQASYAM 54

166 MNTIFKISALTLSAALALSACGKKEAA------SEPAAASAAQGDTSSIGSTMQQASYAM 54

189 MNTIFKISALTLSAALALSACGKKEAA------SEPAAASAAQGDTSSIGSTMQQASYAM 54

215 MNTIFKISALTLSAALALSACGKKEAA------SEPAAASAAQGDTSSIGSTMQQASYAM 54

259 MNTIFKISALTLSAALALSACGKKEAA------SEPAAASAAQGDTSSIGSTMQQASYAM 54

287 MNTIFKISALTLSAALALSACGKKEAA------SEPAAASAAQGDTSSIGSTMQQASYAM 54

366 MNTIFKISALTLSAALALSACGKKEAA------SEPAAASAAQGDTSSIGSTMQQASYAM 54

419 MNTIFKISALTLSAALALSACGKKEAA------SEPTAASAAQGDTSSIGSTMQQASYAM 54

490 MNTIFKISALTLSAALALSACGKKEAA------SEPAAASAAQGDTSSIGSTMQQASYAM 54

589 MNTIFKISALTLSAALALSACGKKEAA------SEPAAASAAQGDTSSIGSTMQQASYAM 54

657 MNTIFKISALTLSAALALSACGKKEAA------SEPAAASAAQGDTSSIGSTMQQASYAM 54

680 MNTIFKISALTLSAALALSACGKKEAA------SEPAAASAAQGDTSSIGSTMQQASYAM 54

526 MNTIFKISALTLSAALALSACSKKEAA------SEPAAASAAQGDTSSIGSTMQQASYAM 54

708 MNTIFKISALTLSAALALSACVKKEAA------SEPAAASAAQGDTSSIGSTMQQASYAM 54

710 MNTIFKISALTLSAALALSACGKKEAA------SEPAAASAAQGDTSSIGSTMQQASYAM 54

1 MNTIFKISALTLSAALALSACGKKEAA------SEPAAASAAQGDTSSIGSTMQQASYAM 54

512 MNTIFKISALTLSAALALSACGKKEAAPAS--ASEPAAASSAQGDTSSIGSTMQQASYAM 58

604 MNTIFKISALTLSAALALSACGKKEAAPAS--ASEPAAASSAQGDTSSIGSTMQQASYAM 58

24 MNKIFKISTLTLAATLALSACGKKEAAPAS--ASEPAAASAAQGDTSSIGSTMQQASYAM 58

116 MNTIFKISALTLSAALALSACGKKEAAPAS--ASEPAAASSAQGDTSSIGSTMQQASYAM 58

359 MNTIFKISALTLSAALALSACGKKEAAPAS--ASEPAAASSAQGDTSSIGSTMQQASYAM 58

653 MNTIFKISALTLSAALALSACGKKEAAPAS--ASEPVAASSAQGDTSSIGSTMQQASYAM 58

568 MNTIFKISALTLSAALALSACGKKEAAPAS--ASEPAAASSAQGDTSSIGSTMQQASYAM 58

13 MNTIFKISALTLSAALALSACGKKEAAPAS--ASEPAAASSAQGDTSSIGSTMQQASYAM 58

257 MNTIFKISALTLSAALALSACGKKEAAPAS--ASEPAAASSAQGDTSSIGSTMQQASYAM 58

** *****:*** .:*.:*** *** . :*.:**: *.:*****. ********

15 GVDIGRSLKQMKEQGAEIDLKVFTEAMQAVYEGKEIKMTEEQAQEVMMKFLQEQQAKAVE 119

319 GVDIGRSLKQMKEQGAEIDLKVFTEAMQAVYDGKEIKMTEEQAQEVMMKFLQEQQAKAVE 114

27 GVDIGRSLKQMKEQGAEIDLKVFTEAMQAVYDGKEIKMTEEQAQEVMMKFLQEQQAKAVE 120

88 GVDIGRSLKQMKEQGAEIDLKVFTEAMQAVYDGKEIKMTEEQAQEVMMKFLQEQQAKAVE 118

113 GVDIGRSLKQMKEQGAEIDLKVFTEAMQAMYDGKEIKMTEEQAQEVMMKFLQEQQAKAVE 119

717 GVDIGRSLKQMKEQGAEIDLKVFTEAMQAVYDGKEIKMTEEQAQEVMMKFLQEQQAKAVE 118

229 GVDIGRSLKQMKEQGAEIDLKVFTEAMQAVYDGKEIKMTEEQAQEVMMKFLQEQQAKAVE 118

324 GVDIGRSLKQMKEQGAEIDLKVFTEAMQAVYEGKEIKMTEEQAQEVMMKFLQEQQAKAVE 119

262 GVDIGRSLKQMKEQGAEIDLKVFTEAMQAVYDGKEIKMTEEQAQEVMMKFLQEQQAKAVE 118

9 GVDIGRSLKQMKEQGAEIDLKVFTEAMQAVYDGKEIKMTEEQAQEVMMKFLQEQQAKAVE 119

295 GVDIGRSLKQMKEQGAEIDLKVFTEAMQAVYDGKEIKMTEEQAQEVMMKFLQEQQAKAVE 114

130 GVDIGRSLKQMKEQGAEIDLKVFTEAMQAVYDGKEIKMTEEQAQEVMMKFLQEQQAKAVE 114

658 GVDIGRSLKQMKEQGAEIDLKVFTEAMQAVYEGKEIKMTEEQAQEVMMKFLQEQQTKAVE 119

58 GVDIGRSLKQMKEQGAEIDLKVFTEAMQAVYEGKEIKMTEEQAQEVMMKFLQEQQAKAVE 119

374 GVDIGRSLKQMKEQGAEIDLKVFTEAMQAVYEGKEIKMTEEQAQEVMMKFLQEQQAKAVE 118

500 GVDIGRSLKQMKEQGAEIDLKVFTEAMQAVYDGKEIKMTEEQAQEVMMKFLQEQQAKAVE 118

10 GVDIGRSLKQMKEQGAEIDLKVFTDAMQAVYDGKEIKMTEEQAQEVMMKFLQEQQAKAVE 118

35 GVDIGRSLKQMKEQGAEIDLKVFTDAMQAVYDGKEIKMTEEQAQEVMMKFLQEQQAKAVE 118

141 GVDIGRSLKQMKEQGAEIDLKVFTEAMQAVYDGKEIKMTEEQAQEVMMKFLQEQQAKAVE 118

628 GVDIGRSLKQMKEQGAEIDLKVFTEAMQAVYDGKEIKMTEEQAQEVMMKFLQEQQAKAVE 118

435 GVDIGRSLKQMKEQGAEIDLKVFTEAMQAVYDGKEIKMTEDQAQEVMMKFLQEQQAKAVE 118

132 GVDIGRSLKQMKEQGAEIDLKVFTEAMQAVYDGKEIKMTEEQAQEVMMKFLQEQQAKAVE 118

356 GVDIGRSLKQMKEQGAEIDLKVFTEAMQAVYDGKEIKMTEEQAQEVMMKFLQEQQAKAVE 118

6 GVDIGRSLKQMKEQGAEIDLKVFTEAMQAVYDGKEIKMTEEQAQEVMMKFLQEQQAKAVE 118

322 GVDIGRSLKQMKEQGAEIDLKVFTEAMQAVYDGKEIKMTEEQAQEVMMKFLQEQQAKAVE 118

301 GVDIGRSLKQMKEQGAEIDLKVFTEAMQAVYDGKEIKMTEEQAQEVMMKFLQEQQAKAVE 118

630 GVDIGRSLKQMKEQGAEIDLKVFTEAMQAVYDGKEIKMTEEQAQEVMMKFLQEQQAKAVE 118

625 GVDIGRSLKQMKEQGAEIDLKVFTEAMQAVYDGKEIKMTEEQAQEVMMKFLQEQQAKAVE 118

179 GVDIGRSLKQMKEQGAEIDLKIFTEAMQAVYDGKEIKMTEEQAQEVMMKFLQEQQAKAVE 118

63 GVDIGRSLKQMKEQGAEIDLKVFTEAMQAVYDGKEIKMTEEQAQEVMMKFLQEQQAKAVE 118

535 GVDIGRSLKQMKEQGAEIDLKVFTEAMQAVYDGKEIKMTEEQAQEVMMKFLQEQQAKAVE 118

125 GVDIGRSLKQMKEQGAEIDLKVFTEAMQAVYDGKEIKMTEEQAQEVMMKFLQEQQAKAVE 118

217 GVDIGRSLKQMKEQGAEIDLKVFTEAMQAVYDGKEIKMTEEQAQEVMMKFLQEQQAKAVE 118

465 GVDIGRSLKQMKEQGAEIDLKVFTEAMQAVYDGKEIKMTEEQAQEVMMKFLQEQQAKAVE 118

66 GVDIGRSLKQMKEQGAEIDLKVFTEAMQAVYDGKEIKMTEEQAQEVMMKFLQEQQAKAVE 118

3 GVDIGRSLKQMKEQGAEIDLKVFTEAMQAVYDGKEIKMTEEQAQEVMMKFLQEQRAKAVE 118

11 GVDIGRSLKQMKEQGAEIDLKVFTEAMQAVYDGKEIKMTEEQAQEVMMKFLQEQQAKAVE 118

453 GVDIGRSLKQMKEQGAEIDLKVFTEAMQAVYDGKEIKMTEEQAQEVMMKFLQEQQAKAVE 118

5 GVDIGRSLKQMKEQGAEIDLKVFTEAMQAVYDGKEIKMTEEQAQEVMMKFLQEQQAKAVE 118

727 GVDIGRSLKQMKEQGAEIDLKVFTEAMQAVYDGKEIKMTEEQAQEVMMKFLQEQQAKAVE 118

686 GVDIGRSLKQMKEQGAEIDLKVFTEAMQAVYDGKEIKMTEEQAQEVMMKFLQEQQAKAVE 118

608 GVDIGRSLKQMKEQGAEIDLKVFTEAMQAVYDGKEIKMTEEQAQEVMMKFLQEQQAKAVE 118

571 GVDIGRSLKQMKEQGAEIDLKVFTEAMQAVYDGKEIKMTEEQAQEVMMKFLQEQQAKAVE 118

554 GVDIGRSLKQMKEQGAEIDLKVFTEAMQAVYDGKEIKMTEEQAQEVMMKFLQEQQAKAVE 118

521 GVDIGRSLKQMKEQGAEIDLKVFTEAMQAVYDGKEIKMTEEQAQEVMMKFLQEQQAKAVE 118

517 GVDIGRSLKQIKEQGAEIDLKVFTEAMQAVYDGKEIKMTEEQAQEVMMKFLQEQQAKAVE 118

507 GVDIGRSLKQMKEQGAEIDLKVFTEAMQAVYDGKEIKMTEEQAQEVMMKFLQEQQAKAVE 118

498 GVDIGRSLKQMKEQGAEIDLKVFTEAMQAVYDGKEIKMTEEQAQEVMMKFLREQQAKAVE 118

486 GVDIGRSLKQMKEQGAEIDLKVFTEAMQAVYDGKEIKMTEEQAQEVMMKFLQEQQAKAVE 118

480 GVDIGRSLKQMKEQGAEIDLKVFTEAMQAVYDGKEIKMTEERAQEVMMKFLQEQQAKAVE 118

466 GVDIGRSLKQMKEQGAEIDLKVFTEAMQAVYDGKEIKMTEEQAQEVMMKFLQEQQAKAVE 118

440 GVDIGRSLKQMQEQGAEIDLKVFTEAMQAVYDGKEIKMTEEQAQEVMMKFLQEQQAKAVE 118

439 GVDIGRSLKQMKEQGVEIDLKVFTEAMQAVYDGKEIKMTEEQAQEVMMKFLQEQQAKAVE 118

370 GVDIGRSLKQMKEQGAEIDLKVFTEAMQAVYDGKEIKMTEEQAQEVMMKFLQEQQAKAVE 118

367 GVDIGRSLKQMKEQGAEIDLKVFTEAMQAVYDGKEIKMTEEQAQEVMMKFLQEQQAKAVE 118

219 GVDIGRSLKQMKEQGAEIDLKVFTEAMQAVYDGKEIKMTEEQAQEVMMKFLQEQQAKAVE 118

197 GVDIGRSLKQMKEQGAEIDLKVFIEAMQAVYDGKEIKMTEEQAQEVMMKFLQEQQAKAVE 118

494 GVDIGRSLKQMKEQGAEIDLKVFTEAMQAVYDGKEIKMTEEQAQEVMMKFLQEQQAKAVE 118

196 GVDIGRSLKQMKEQGAEIDLKVFTEAMQAVYDGKEIKMTEEQAQEVMMKFLQEQQAKAVE 118

182 GVDIGRSLKQMKEQGAEIDLKVFTEAMQAVYDGKEIKMTEEQAQEVMMKFLQEQQAKAVE 118

265 GVDIGRSLKQMKEQGAEIDLKVFTEAMQAVYDGKEIKMTEEQAQEVMMKFLQEQQAKAVE 118

155 GVDIGRSLKQMKEQGAEIDLKVFTEAMQAVYDGKEIKMTEEQAQEVMMKFLQEQQAKAVE 118

114 GVDIGRSLKQMKEQGAEIDLKVFTEAMQAVYGGKEIKMTEEQAQEVMMKFLQEQQAKAVE 118

112 GVDIGRSLKQMKEQGAEIDLKVFTEAMQAVYDGKEIKMTEEQAQEVMMKFFQEQQAKAVE 118

110 GVDIGRSLKQMKEQGAEIDLKVFTEAMQAVYDGKEIKMTEEQAQEVMMKFLQEQQAKAVE 118

74 GVDIGHSLKQMKEQGAEIDLKVFTEAMQAVYDGKEIKMTEEQAQEVMMKFLQEQQAKAVE 118

659 GVDIGRSLKQMKEQGAEIDLKVFTEAMQAVYDGKEIKMTEEQAQEVMMKFLQEQQAKAVE 118

627 GVDIGRSLKQMKEQGAEIDLKVFTEAMQAVYDGKEIKMTEEQAQEVMMKFLQEQQAKAVE 118

502 GVDIGRSLKQMKEQGAEIDLKVFTEAMQAVYDGKEIKMTEEQAQEVMMKFLQEQQAKAVE 118

371 GVDIGRSLKQMKEQGAEIDLKVFTEAMQAVYDGKEIKMTEEQAQEVMMKFLQEQQAKAVE 118

320 GVDIGRSLKQMKEQGAEIDLKVFTEAMQAVYDGKEIKMTEEQAQEVMMKFLQEQQAKAVE 118

290 GVDIGRSLKQMKEQGAEIDLKVFTEAMQAVYDGKEIKMTEEQAQEVMMKFLQEQQAKAVE 118

216 GVDIGRSLKQMKEQGAEIDLKVFTEAMQAVYDGKEIKMTEEQAQEVMMKFLQEQQAKAVE 118

146 GVDIGRSLKQMKEQGAEIDLKVFTEAMQAVYDGKEIKMTEEQAQEVMMKFLQEQQAKAVE 118

109 GVDIGRSLKQMKEQGAEIDLKVFTEAMQAVYDGKEIKMTEEQAQEVMMKFLQEQQAKAVE 118

572 GVDIGRSLKQMKEQGAEIDLKVFTEAMQAVYDGKEIKMTEEQAQEVMMKFLQEQQAKAVE 118

540 GVDIGRSLKQMKEQGAEIDLKVFTEAMQAVYDGKEIKMTEEQAQEVMMKFLQEQQAKAVE 118

365 GVDIGRSLKQMKEQGAEIDLKVFTEAMQAVYDSKEIKMTEEQAQEVMMKFLQEQQAKAVE 118

148 GVDIGRSLKQMKEQGAEIDLKVFTEAMQAVYDGKEIKMTEEQAQEVMMKFLQEQQAKAVE 118

726 GVDIGRSLKQMKEQGAEIDLKVFTEAMQAVYDGKEIKMTEEQAQEVMMKFLQEQQVKAVE 118

729 GVDIGRSLKQMKEQGAEIDLKVFTEAMQAVYDGKEIKMTEEQAQEVMMKFLQEQQAKAVE 118

331 GVDIGRSLKQMKEQGAEIDLKVFTEAMQAVYDGKEIKMTEEQAQEVMMKFLQEQQAKAVE 118

396 GVDIGRSLKQMKEQGAEIDLKVFTEAMQAVYDGKEIKMTEEQAQEVMMKFLQEQQAKAVE 118

406 GVDIGRSLKQMKEQGAEIDLKVFTEAMQAVYDGKEIKMTEEQAQEVMMKFLQEQQAKAVE 118

438 GVDIGRSLKQMKEQGAEIDLKVFTEAMQAVYDGKEIKMTEEQAQEVMMKFLQEQQAKAVE 118

483 GVDIGRSLKQMKEQGAEIDLKVFTEAMQAVYDGKEIKMTEEQAQEVMMKFLQEQQAKALE 118

570 GVDIGRSLKQMKEQGAEIDLKVFTEAMQAVYDGKEIKMTEEQAQEVMMKFLQEQQAKAVE 118

655 GVDIGRSLKQMKEQGAEIDLKVFTEAMQAVYDGKEIKMTEEQAQEVMMKFLQEQQAKAVE 118

716 GVDIGRSLKQMKEQGAEIDLKVFTEAMQAVYDGKEIKMTEEQAQEVMMKFLQEQQAKAVE 118

147 GVDIGRSLKQMKEQGAEIDLKVFTEAMQAVYDGKEIKMTEEQAQEVMMKFLQEQQAKAVE 118

321 GVDIGRSLKQMKEQGAEIDLKVFTEAMQAVYDGKEIKMTEEQAQEVMMKFLQEQQAKAVE 118

688 GVDIGRSLKQMKEQGAEIDLKVFTEAMQAVYDGKEIKMTEEQAQEVMMKFLQEQQAKAVE 118

299 GVDIGRSLKQMKEQGAEIDLKVFTEAMQAVYDGKEIKMTEEQAQEVMMKFLQEQQAKAVE 118

622 GVDIGRSLKQMKEQGAEIDLKVFTEAMQAVYDGKEIKMTEEQAQEVMMKFLQEQQAKAVE 118

405 GVDIGRSLKQMKEQGAEIDLKVFTEAMQAVYDGKEIKMTEEQAQEVMMKFLQEQQAKAVE 118

528 GVDIGRSLKQMKEQGAEIDLKVFTEAMQAVYDGKEIKMTEEQAQEVMMKFLQEQQAKAVE 118

94 GVDIGRSLKQMKEQGAEIDLKVFTEAMQAVYDGKEIKMTEEQAQEVMMKFLQEQQAKAVE 118

399 GVDIGRSLKQMKEQGAEIDLKVFTEAMQAVYDGKEIKMTEEQAQEVMMKFLQEQQAKAVE 118

2 GVDIGRSLKQMKEQGAEIDLKVFTEAMQAVYDGKEIKMTEEQAQEVMMKFLQEQQAKAVE 118

152 GVDIGRSLKQMKEQGAEIDLKVFTEAMQAVYDGKEIKMTEEQAQEVMMKFLQEQQAKAVE 118

415 GVDIGRSLKQMKEQGAEIDLKVFTEAMQAVYDGKEIKMTEEQAQEVMMKFLQEQQAKAVE 118

323 GVDIGRSLKQMKEQGAEIDLKVFTEAMQAVYDGKEIKMTEEQAQEVMMKFLQEQQAKAVE 118

96 GVDIGRSLKQMKEQGAEIDLKVFTEAMQAVYDGKEIKMTEEQAQEVMMKFLQEQQAKAVE 118

529 GVDIGRSLKQMKEQGAEIDLKVFTEAMQAVYDGKEIKMTEEQAQEVMMKFLQEQQAKAVE 118

541 GVDIGRSLKQMKEQGAEIDLKVFTEAMQAVYDGKEIKMTEEQAQEVMMKFLQEQQAKAVE 118

687 GVDIGRSLKQMKEQGAEIDLKVFTEAMQAVYEGKEIKMTEEQAQEVMMKFLQEQQAKAVE 118

107 GVDIGRSLKQMKEQGAEIDLKVFTEAMQAVYDGKEIKMTEEQAQEVMMKFLQEQQAKAVE 114

266 GVDIGRSLKQMKEQGAEIDLKVFAEAMQAVYDGKEIKMTEEQAQEVMMKFLQEQQAKAVE 114

545 GVDIGRSLKQMKEQGAEIDLKVFTEAMQAVYDGKEIKMTEEQAQEVMMKFLQEQQAKAVE 114

685 GVDIGRSLKQMKEQGAEIDLKVFTEAMQAVYDGKEIKMTEEQAQEVMMKFLQEQQAKAVE 114

7 GVDIGRSLKQMKEQGAEIDLKVFTEAMQAVYDGKEIKMTEEQAQEVMMKFLQEQQAKAVE 114

549 GVDIGRSLKQMKEQGAEIDLKVFTEAMQAVYDGKEIKMTEEQAQEVMMKFLQEQQAKAVE 114

553 GVDIGRSLKQMKEQGAEIDLKVFTEAMQAVYDGKEIKMTEEQAQEVMMKFLQEQQAKAVE 114

222 GVDIGRSLKQMKEQGAEIDLKVFTEAMQAVYDGKEIKMTEEQAQEVMMKFLQEQQAKAVE 114

157 GVDIGRSLKQMKEQGAEIDLKVFTEAMQAVYDGKEIKMTEEQAQEVMMKFLQEQQAKAVE 114

294 GVDIGRSLKQMKEQGAEIDLKVFTEAMQAVYDGKEIKMTEEQAQEVMMKFLQEQQAKAVE 114

441 GVDIGRSLKQMKEQGAEIDLKVFTEAMQAMYDGKEIKMTEEQAQEVMMKFLQEQQAKAVE 114

654 GVDIGRSLKQMKEQGAEIDLKVFTEAMQAVYDGKEIKMTEEQAQEVMMKFLQEQQAKAVE 114

736 GVDIGRSLKQMKELGAEIDLKVFTEAMQAVYDGKEIKMTEEQAQEVMMKFLQEQQAKAVE 114

240 GVDIGRSLKQMKEQGAEIDLKVFTEAMQAVYDGKEIKMTEEQAQEVMMKFLQEQQAKAVE 114

22 GVDIGRSLKQMKEQGAEIDLKVFTEAMQAVYDGKEIKMTEEQAQEVMMKFLQEQQAKAVE 114

188 GVDIGRSLKQMKEQGAEIDLKVFTEAMQAVYDGKEIKMTEEQAQEVMMKFLQEQQAKAVE 114

638 GVDIGRSLKQMKEQGAEIDLKVFTEAMQAVYDGKEIKMTEEQAQEVMMKFLQEQQAKAVE 114

106 GVDIGRSLKQMKEQGAEIDLKVFTEAMQAVYDGKEIKMTEEQAQEVMMKFLQEQQAKAVE 114

105 GVDIGRSLKQMKEQGAEIDLKVFTEAMQAVYDGKEIKMTEEQAQEVMMKFLKEQQAKAVE 114

166 GVDIGRSLKQMKEQGAEIDLKVFTEAMQAVYDGKEIKMTEEQAQEVMMKFLQEQQAKAVE 114

189 GVDIGRSLKQMKEQGAEIDLKVFTEAMQAVYDGKEIKMTEEQAQEVMMKFLQEQQAKAVE 114

215 GVDIGRSLKQMKEQGAEIDLKVFTEAMQAVYDGKEIKMTEEQAQEVMMKFLQEQQAKAVE 114

259 GVDIGRSLKQMKEQGAEIDLKVFTEAMQAVYDGKEIKITEEQAQEVMMKFLQEQQAKAVE 114

287 GVDIGRSLKQMKEQGAEIDLKVFTEAMQAVYDGKEIKMTEEQAQEVMMKFLQEQQAKAVE 114

366 GVDIGRSLKQMKEQGAEIDLKVFTEAMQAVYDGKEIKMTEEQAQEVMMKFLQEQQAKAVE 114

419 GVDIGRSLKQMKEQGAEIDLKVFTEAMQAVYDGKEIKMTEEQAQEVMMKFLQEQQAKAVE 114

490 GVDIGRSLKQMKEQGAEIDLKVFTEAMQAVYDGKEIKMTEEQAQEVMMKFLQEQQAKAVE 114

589 GVDIGRSLKQMKEQGAEIDLKVFTEAMQAVYDGKEIKMTEEQAQEVMMKFLQEQQAKAVE 114

657 GVDIGRSLKQMKEQGAEIDLKVFTEAMQAVYDGKEIKMTEEQAQEVMMKFLQEQQAKAVE 114

680 GVDIGRSLKQMKEQGAEIDLKVFTEAMQAVYDGKEIKMTEEQAQEVMMKFFQEQQAKAVE 114

526 GVDIGRSLKQMKEQGAEIDLKVFTEAMQAVYDGKEIKMTEEQAQEVMMKFLQEQQAKAVE 114

708 GVDIGRSLKQMKEQGAEIDLKVFTEAMQAVYDGKEIKMTEEQAQEVMMKFLQEQQAKAVE 114

710 GVDIGRSLKQMKEQGAEIDLKVFTEAMQAVYDGKEIKMTEEQAQEVMMKFLQEQQAKAVE 114

1 GVDIGRSLKQMKEQGAEIDLKVFTEAMQAVYDGKEIKMTEEQAQEVMMKFLQEQQAKAVE 114

512 GVDIGRSLKQMKEQGAEIDLKVFTEAMQAVYDGKEIKMTEEQAQEVMMKFLQEQQAKAVE 118

604 GVDIGRSLKQMKEQGAEIDLKVFTEAMQAVYDGKEIKMTEEQAQEVMMKLLREQQAKAVE 118

24 GVDIGRSLKQMKEQGAEIDLKVFTEAMQAVYDGKEIKMTEEQAQEVMMKFLQEQQAKAVE 118

116 GVDIGRSLKQMKEQGAEIDLKVFTEAMQAVYDGKEIKMTEEQAQEVMMKFLQEQQAKAVE 118

359 GVDIGRSLKQMKEQGAEIDLKVFTEAMQAVYDGKEIKMTEDQAQEVMMKFLQEQQAKAVE 118

653 GVDIGRSLKQMKEQGAEIDLKVFTEAMQAVYDGKEIKMTEEQAQEVMMKFLQEQQAKAVE 118

568 GVDIGRSLKQMKEQGAEIDLKVFTEAMQAVYDGKEIKMTEEQAQEVMMKFLQEQQAKAVE 118

13 GVDIGRSLKQMKEQGAEIDLKVFTEAMQAVYDGKEIKMTEEQAQEVMMKFLQEQQAKAVE 118

257 GVDIGRSLKQMKEQGAEIDLKVFTEAMQAVYDGKEIKMTEEQAQEVMMKFLQEQQAKAVE 118

*****:****::* *.*****:* :****:* .****:**::*******:::**:.**:*

15 KHKADAKANKEKGEAFLKENAAKEGVKTTASGLQYKITKQGEGKQPTKDDIVTVEYEGRL 179

319 KHKADAKANKEKGEAFLKENAGKESVKTTASGLQYKITKQGEGKQPTKDDIVTVEYEGRL 174

27 KHKADAKANKEKGEAFLKENAGKEGVKTTASGLQYKITKQGEGKQPTKDDIVTVEYEGRL 180

88 KHKADAKANKEKGEAFLKENAAKEGVKTTASGLQYKITKQGEGKQPTKDDIVTVEYEGRL 178

113 KHKADAKANKEKGEAFLKENAAKDGVKTTASGLQYKITKQGEGKQPTKDDIVTVEYEGRL 179

717 KHKADAKANKEKGEAFLKENAAKEGVKTTASGLQYKITKQGEGKQPTKDDIVTVEYEGRL 178

229 KHKADAKANKEKGEAFLKENAAKEGVKTTASGLQYKITKQGEGKQPTKDDIVTVEYEGRL 178

324 KHKADAKANKEKGEAFLKENASKDGVKTTASGLQYKITKQGEGKQPTKDDIVTVEYEGRL 179

262 KHKADAKANKEKGEAFLKENAAKDGVKTTASGLQYKITKQGEGKQPTKDDIVTVEYEGRL 178

9 KHKADAKANKEKGEAFLKENAAKEGVKTTASGLQYKITKQGEGKQPTKDDIVTVEYEGRL 179

295 KHKADAKANKEKGEAFLKENAAKDGVKTTASGLQYKITKQGEGKQPTKDDIVTVEYEGRL 174

130 KHKAEAKANKEKGEAFLKENAAKDGVKTTASGLQYKITKQGEGKQPTKDDIVTVEYEGRL 174

658 KHKADAKANKEKGEAFLKENAAKEGVKTTASGLQYKITKQGEGKQPTKDDIVTVEYEGRL 179

58 KHKADAKANKEKGEAFLKENAAKEGVKTTASGLQYKITKQGEGKQPTKDDIVTVEYEGRL 179

374 KHKADAKANKEKGEAFLKENAAKEGVKTTASGLQYKITKQGEGKQPTKDDIVTVEYEGRL 178

500 KHKADAKANKEKGEAFLKENAAKDGVKTTASGLQYKITKQGEGKQPTKDDIVTVEYEGRL 178

10 KHKADAKANKEKGEAFLKENAAKDGVKTTASGLQYKITKQGKGKQPTKDDIVTVEYEGRL 178

35 KHKADAKANKEKGEAFLKENAAKDGVKTTASGLQYKITKQGEGKQPTKDDIVTVEYEGRL 178

141 KHKADAKANKEKGEAFLKENAAKDGVKTTASGLQYKITKQGEGKQPSKDDIVTVEYEGRL 178

628 KHKADAKANKEKGEAFLKENAAKDGVKTTASGLQYKITKQGEGKQPSKDDIVTVEYEGRL 178

435 KHKADAKANKEKGEAFLKENAAKDGVKTTASGLQYKITKQGEGKQPTKDDIVTVEYEGRL 178

132 KHKADAKANKEKGEAFLKENAAKDGVKTTASGLQYKITKQGEGKQPTKDDIVTVEYEGRL 178

356 KHKADAKANKEKGEAFLKENAAKDGVKTTASGLQYKITKQGEGKQPTKDDIVTVEYEGRL 178

6 KHKADAKANKEKGEAFLKENAGKESVKTTASGLQYKITKQGEGKQPTKDDIVTVEYEGRL 178

322 KHKADAKANKEKGEAFLKENAGKESVKTTASGLQYKITKQGEGKQPTKDDIVTVEYEGRL 178

301 KHKADAKANKEKGEAFLKENAAKDGVKTTASGLQYKITKQGEGKQPTKDDIVTVEYEGRL 178

630 KHKADAKANKEKGEAFLKENAAKDGVKTTASGLQYKITKQGEGKQPTKDDIVTVEYEGRL 178

625 KHKADAKANKEKGEAFLKENAAKDGVKTTASGLQYKITKQGEGKQPTKDDIVTVEYEGRL 178

179 KHKADAKANKEKGEAFLKENAAKDGVKTTASGLQYKITKQGEGKQPTKDDIVTVEYEGRL 178

63 KHKADAKANKEKGEAFLKENAAKDGVKTTASGLQYKITKQGEGKQPSKDDIVTVEYEGRL 178

535 KHKADAKANKEKGEAFLKENAAKDGVKTTASGLQYKITKQGEGKQPSKDDIVTVEYEGRL 178

125 KHKADAKANKEKGEAFLKENAAKDGVKTTASGLQYKITKQGEGKQPTKDDIVTVEYEGRL 178

217 KHKADAKANKEKGEAFLKENAAKDGVKTTASGLQYKITKQGEGKQPTKDDIVTVEYEGRL 178

465 KHKAEAKANKEKGEAFLKENAAKDGVKTTASGLQYKITKQGEGKQPTKDDIVTVEYEGRL 178

66 KHKADAKANKEKGEAFLKENAAKDGVKTTASGLQYKITKQGEGKQPSKDDIVTVEYEGRL 178

3 KHKADAKANKEKGEAFLKENAAKDGVKTTASGLQYKITKQGEGKQPTKDDIVTVEYEGRL 178

11 KHKADAKANKEKGEAFLKENAAKDGVKTTASGLQYKITKQGEGKQPTKDDIVTVEYEGRL 178

453 KHKADAKANKEKGEAFLKENAAKDGVKTTASGLQYKITKQGEGKQPTKDDIVTVEYEGRL 178

5 KHKADAKANKEKGEAFLKENAAKDGVKTTASGLQYKITKQGEGKQPTKDDIVTVEYEGRL 178

727 KHKADAKANKEKGEAFLKENAAKDGVKTTASGLQYKITKQGEGKQPTKDDIVTVEYEGRL 178

686 KHKADAKANKEKGEAFLKENAAKDGVKTTASGLQYKITKQGEGKQPTKDDIVTVEYEGRL 178

608 KHKADAKANKEKGEAFLKENAAKDGVKTTASGLQYKITKQGEGKQPTKDDIVTVEYEGRL 178

571 KHKADAKANKEKGEAFLKENAAKDGVKTTASGLQYKITKQGEGKQPTKDDIVTVEYEGRL 178

554 KHKADAKANKEKGEAFLKENAAKDGVKTTASGLQYKITKQGEGKQPTKDDIVTVEYEGRL 178

521 KHKADAKANKEKGEAFLKENAAKDGVKTTVSGLQYKITKQGEGKQPTKDDIVTVEYEGRL 178

517 KHKADAKANKEKGEAFLKENAAKDGVKTTASGLQYKITKQGEGKQPTKDDIVTVEYEGRL 178

507 KHKADAKANKEKGEAFLKENAAKDGVKTTASGLQYKITKQGEGKQPTKDDIVTVEYEGRL 178

498 KHKADAKANKEKGEAFLKENAAKDGVKTTASGLQYKITKQGEGKQPTKDDIVTVEYEGRL 178

486 KHKVDAKANKEKGEAFLKENAAKDGVKTTASGLQYKITKQGEGKQPTKDDIVTVEYEGRL 178

480 KHKADAKANKEKGEAFLKENAAKDGVKTTASGLQYKITKQGEGKQPTKDDIVTVEYEGRL 178

466 KHKADAKANKEKGEAFLKENAAKDGVKTTASGLQYKITKQGEGKQPTKDDIVTVEYEGRL 178

440 KHKADAKANKEKGEAFLKENAAKDGVKTTASGLQYKITKQGEGKQPTKDDIVTVEYEGRL 178

439 KHKADAKANKEKGEAFLKENAAKDGVKTTASGLQYKITKQGEGKQPTKDDIVTVEYEGRL 178

370 KHKADAKANKEKGEAFLKENAAKDGVKTTASGLQYKITKQGEGKQPTKDDIVTVEYEGRL 178

367 KHKADAKANKEKGEAFLKENVAKDGVKTTASGLQYKITKQGEGKQPTKDDIVTVEYEGRL 178

219 KHKADAKANKEKGEAFLKENAAKDGVKTTASGLQYKITKQSEGKQPTKDDIVTVEYEGRL 178

197 KHKADAKANKEKGEAFLKENAAKDGVKTTASGLQYKITKQGEGKQPTKDDIVTVEYEGRL 178

494 KHKADAKANKEKGEAFLKENAAKDGVKTTASGLQYKITKQGEGKQPTKDDIVTVEYEGRL 178

196 KHKADAKANKEKGEAFLKENAAKDGVKTTASGLQYKITKQGEGKQPTKDDIVTVEYEGRL 178

182 KHKADAKANKEKGEAFLKENAAKDGVKTTASGLQYKITKQGEGKQPTKDDIVTVEYEGRL 178

265 KHKADAKANKEKGEAFLKENAAKDGVKTTASGLQYKITKQGEGKQPTKDDIVTVEYEGRL 178

155 KHKADAKANKEKGEAFLKENAAKDGVKTTASGLQYKITKQGEGKQPTKDDIVTVEYEGRL 178

114 KHKADAKANKEKGEAFLKENAAKDGVKTTASGLQYKITKQGEGKQPTKDDIVTVEYEGRL 178

112 KHKADAKANKEKGEAFLKENAAKDGVKTTASGLQYKITKQGEGKQPTKDDIVTVEYEGRL 178

110 KHKADTKANKEKGEAFLKENAAKDGVKTTASGLQYKITKQGEGKQPTKDDIVTVEYEGRL 178

74 KHKADAKANKEKGEAFLKENAAKDGVKTTASGLQYKITKQGEGKQPTKDDIVTVEYEGRL 178

659 KHKADAKANKEKGEAFLKENAAKDGVKTTASGLQYKITKQGEGKQPTKDDIVTVEYEGRL 178

627 KHKADAKANKEKGEAFLKENAAKDGVKTTASDLQYKITKQGEGKQPTKDDIVTVEYEGRL 178

502 KHKADAKANKEKGEAFLKENAAKDGVKTTASGQQYKITKQGEGKQPTKDDIVTVEYEGRL 178

371 KHKADAKANKEKGEAFLKENAAKDGVKTTASGLQYKITKQGEGKQPTKDDIVTVEYEGRL 178

320 KHKADAKANKEKGEAFLKENAAKDGVKTTASGLQYKITKQGEGKQPTKDDIVTVEYEGRL 178

290 KYKADAKANKEKGEAFLKENAAKDGVKTTASGLQYKITKQGEGKQPTKDDIVTVEYEGRL 178

216 KHKADAKANKEKGEAFLKENAAKDGVKTTASGLQYKITKQGEGKQPTKDDIVTVEYEGRL 178

146 KHKADAKANKEKGEAFLKENAAKDGVKTTASGLQYKITKQGEGKQPTKDDIVTVEYEGRL 178

109 KHKADAKANKEKGEAFLKENAAKDGVKTTASGLQYKITKQGEGKQPTKDDIVTVEYEGRL 178

572 KHKADAKANKEKGEAFLKENAAKDGVKTTASGLQYKITKQGEGKQPTKDDIVTVEYEGRL 178

540 KHKADAKANKEKGEAFLKENAAKDGVKTTASGLQYKITKQGEGKQPTKDDIVTVEYEGRL 178

365 KHKADAKANKEKGEAFLKENAAKDGVKTTASGLQYKITKQGEGKQPTKDDIVTVEYEGRL 178

148 KHKADAKANKEKGEAFLKENAAKDGVKTTASGLQYKITKQGEGKQPTKDDIVTVEYEGRL 178

726 KHKADAKANKEKGEAFLKENAAKDGVKTTASGLQYKITKQGEGKQPTKDDIVTVEYEGRL 178

729 KHKADAKANKEKGEAFLKENAAKDGVKTTASGLQYKITKQGEGKQLTKDDIVTVEYEGRL 178

331 KHKADAKANKEKGEAFLKENAGKDGVKTTASGLQYKITKQGEGKQPTKDDIVTVEYEGRL 178

396 KHKADAKANKEKGEAFLKENAAKDGVKTTASGLQYKITKQGEGKQPTKDDIVTVEYEGRL 178

406 KHKADAKANKEKGKAFLKENAAKDGVKTTASGLQYKITKQGEGKQPTKDDIVTVEYEGRL 178

438 KHKADAKANKEKGEAFLKENAAKDGVKTTASGLQYKITKQGEGKQPTKDDIVTVEYEGRL 178

483 KHKADAKANKEKGEAFLKENAAKDGVKTTASGLQYKITKQGEGKQPTKDDIVTVEYEGRL 178

570 KHKADAKANKEKGEAFLKENAAKDGVKTTASGLQYKITKQGEGKQPTKDDIVTVEYEGHL 178

655 KHKADAKANKEKGEAFLKENAAKDGVKTTASGLQYKITKQGEGKQPTKDDIVTVEYEGRL 178

716 KHKADAKANKEKGEAFLKENAAKDGVKTTASGLQYKITKQGEGKQPTKDDIVTVEYEGRL 178

147 KHKADAKANKEKGEAFLKENAAKDGVKTTASGLQYKITKQGEGKQPTKDDIVTVEYEGRL 178

321 KHKADAKANKEKGEAFLKENAAKDGVKTTASGLQYKITKQGEGKQPTKDDIVTVEYEGRL 178

688 KHKADAKANKEKGEAFLKENAAKDGVKTTASGLQYKITKQGEGKQPTKDDIVTVEYEGRL 178

299 KHKADAKANKEKGEAFLKENAAKDGVKTTASGLQYKITKQGEGKQPTKDDIVTVEYEGRL 178

622 KHKADAKANKEKGEAFLKENAAKDGVKTTASGLQYKITKQGEGKQPTKDDIVTVEYEGRL 178

405 KHKADAKANKEKGEAFLKENAAKDGVKTTASGLQYKITKQGEGKQPTKDDIVTVEYEGRL 178

528 KHKADAKANKEKGEAFLKENAAKDGVKTTASGLQYKITKQGEGKQPTKDDIVTVEYEGRL 178

94 KHKADAKANKEKGEAFLKENAAKDGVKTTASGLQYKITKQGEGKQPTKDDIVTVEYEGRL 178

399 KHKADAKANKEKGEAFLKENAAKDGVKTTASGLQYKITKQGEGKQPTKDDIVTVEYEGRL 178

2 KHKADAKANKEKGEAFLKENAAKDGVKTTASGLQYKITKQGEGKQPTKDDIVTVEYEGRL 178

152 KHKADAKANKEKGEAFLKENAAKDGVKTTASGLQYKITKQGEGKQPTKDDIVTVEYEGRL 178

415 KHKADAKANKEKGEAFLKENAAKDGVKTTASGLQYKITKQGEGKQPTKDDIVTVEYEGRL 178

323 KHKADAKANKEKGEAFLKENAAKDGVKTTASGLQYKITKQGEGKQPTKDDIVTVEYEGRL 178

96 KHKADAKANKEKGEAFLKENAAKDGVKTTASGLQYKITKQGKGKQPTKDDIVTVEYEGRL 178

529 KHKADAKANKEKGEAFLKENAAKDGVKTTASGLQYKITKQGEGKQPTKDDIVTVEYEGRL 178

541 KHKADAKANKEKGEAFLKENAAKEGVKTTASGLQYKITKQGEGKQPTKDDIVTVEYEGRL 178

687 KHKADAKANKEKGEAFLKENAAKEGVKTTASGLQYKITKQGEGKQPTKDDIVTVEYEGRL 178

107 KHKADAKANKEKGEAFLKENAGKESVKTTASGLQYKITKQGEGKQPTKDDIVTVEYEGRL 174

266 KHKAEAKANKEKGEAFLKENAAKDGVKTTASGLQYKITKQGEGKQPTKDDIVTVEYEGRL 174

545 KHKAEAKANKEKGEAFLKENAAKDGVKTTASGLQYKITKQGEGKQPTKDDIVTVEYEGRL 174

685 KHKAEAKANKEKGEAFLKENAAKDGVKTTASGLQYKITKQGEGKQPTKDDIVTVEYEGRL 174

7 KHKAEAKANKEKGEAFLKENAAKDGVKTTASGLQYKITKQGEGKQPTKDDIVTVEYEGRL 174

549 KHKADAKANKEKGEAFLKENAAKDGVKTTASGLQYKITKQGEGKQPTKDDIVTVEYEGRL 174

553 KHKADAKANKEKGEAFLKENAAKDGVKTTASGLQYKITKQGEGKQPSKDDIVTVEYEGRL 174

222 KHKADAKANKEKGEAFLKENAAKDGVKTTASGLQYKITKQSEGKQPTKDDIVTVEYEGRL 174

157 KHKADAKANKEKGEAFLKENAAKDGVKTTASGLQYKITKQGEGKQPTKDDIVTVEYEGRL 174

294 KHKADAKANKEKGEAFLKENAAKDGVKTTASGLQYKITKQGEGKQPTKDDIVTVEYEGRL 174

441 KHKADAKANKEKGEAFLKENAAKDGVKTTASGLQYKITKQGEGKQPTKDDIVTVEYEGRL 174

654 KHKADAKANKEKGEAFLKENAAKDGVKTTASGLQYKITKQGEGKQPTKDDIVTVEYEGRL 174

736 KHKADAKANKEKGEAFLKENAAKDGVKTTASGLQYKITKQGEGKQPTKDDIVTVEYEGRL 174

240 KHKADAKANKEKGEAFLKENAAKDGVKTTASGLQYKITKQGEGKQPTKDDIVTVEYEGRL 174

22 KHKADAKANKEKGEAFLKENAAKDGVKTTASGLQYKITKQGEGKQPTKDDIVTVEYEGRL 174

188 KHKADAKANKEKGEAFLKENAAKDGVKTTASGLQYKITKQSEGKQPSKDDIVTVEYEGRL 174

638 KHKADAKANKEKGEAFLKENAAKDGVKTTASGLQYKITKQGEGKQPSKDDIVTVEYEGRL 174

106 KHKADAKANKEKGEAFLKENAAKDGVKTTASGLQYKITKQGEGKQPSKDDIVTVEYEGRL 174

105 KHKADAKANKEKGEAFLKENAAKDGVKTTASGLQYKITKQGEGKQPSKDDIVTVEYEGRL 174

166 KHKADAKANKEKGEAFLKENAAKDGVKTTASGLQYKITKQGESKQPSKDDIVTVEYEGRL 174

189 KHKADAKANKEKGEAFLKENTAKDGVKTTASGLQYKITKQGEGKQPSKDDIVTVEYEGRL 174

215 KHKADAKANKEKGEAFLKENAAKDGVKTTASGLQYKITKQGEGKQPSKDDIVTVEYEGRL 174

259 KHKADAKANKEKGEAFLKENAAKDGVKTTASGLQYKITKQGEGKQPSKDDIVTVEYEGRL 174

287 KHKADAKANKEKGEAFLKENAAKDGVKTTASGLQYKITKQGEGKQPSKDDIVTVEYEGRL 174

366 KHKADAKANKEKGEAFLKENAAKDGVKTTASGLQYKITKQGEGKQPSKDDIVTVEYEGRL 174

419 KHKADAKANKEKGEAFLKENAAKDGVKTTASGLQYKITKQGEGKQPSKDDIVTVEYEGRL 174

490 KHKADAKANKEKGEAFLKENAAKDGVKTTASGLQYKITKQGEGKQPSKDDIVTVEYEGRL 174

589 KHKADAKANKEKGEAFLKENAAKDGVKTTASGLQYKITKQGEGKQPSKDDIVTVEYEGRL 174

657 KHKADAKANKEKGEAFLKENAAKDGVKTTASGLQYKITKQGEGKQPSKDDIVTVEYEGRL 174

680 KHKADAKANKEKGEAFLKENAAKDGVKTTASGLQYKITKQGEGKQPSKDDIVTVEYEGRL 174

526 KHKADAKANKEKGEAFLKENAAKDGVKTTASGLQYKITKQGEGKQPSKDDIVTVEYEGRL 174

708 KHKADAKANKEKGEAFLKENAAKDGVKTTASGLQYKITKQGEGKQPSKDDIVTVEYEGRL 174

710 KHKADAKANKEKGEAFLKENAAKDGVKTTASGLQYKITKQGEGKQPSKDDIVTVEYEGRL 174

1 KHKADAKANKEKGEAFLKENAAKDGVKTTASGLQYKITKQGEGKQPSKDDIVTVEYEGRL 174

512 KHKADAKANKEKGEAFLKENAAKDGVKTTASGLQYKITKQGEGKQPSKDDIVTVEYEGRL 178

604 KHKSDAKANKEKGEAFLKENAAKDGVKTTASGLQYKITKQGEGKQPTKDDIVTVEYEGRL 178

24 KHKADAKANKEKGEAFLKENAAKDGVKTTASGLQYKITKQGEGKQPTKDDIVTVEYEGRL 178

116 KHKADAKANKEKGEAFLKENAAKDGVKTTASGLQYKITKQGEGKQPTKDDIVTVEYEGRL 178

359 KHKADAKANKEKGEAFLKENAGKESVKTTASGLQYKITKQGEGKQPTKDDIVTVEYEGRL 178

653 KHKADAKANKEKGEAFLKENAAKDGVKTTASGLQYKITKQGEGKQPTKDDIVTVEYEGRL 178

568 KHKADAKANKEKGEAFLKENAAKDGVKTTASGLQYKITKQGEGKQPSKDDIVTVEYEGRL 178

13 KHKADAKANKEKGEAFLKENAAKDGVKTTASGLQYKITKQGEGKQPTKDDIVTVEYEGRL 178

257 KHKADAKANKEKGEAFLKENAAKDGVKTTASGLQYKITKQGEGKQPTKDDIVTVEYEGRL 178

*:* ::*******:******..*:.****.*. *******.:.** :***********:*

15 IDGTVFDSSKANGGPATFPLSQVIPGWTEGVQLLKEGGEATFYIPSNLAYREQGAGEKIG 239

319 IDGTVFDSSKANGGPATFPLSQVIPGWTEGVQLLKEGGEATFYIPSNLAYREQGAGEKIG 234

27 IDGTVFDSSKANGGPATFPLSQVIPGWTEGVQLLKEGGEATFYIPSNLAYREQGAGEKIG 240

88 IDGTVFDSSKANGGPATFPLSQVIPGWTEGVQLLKEGGEATFYIPSNLAYREQGAGEKIG 238

113 IDGTVFDSSKANGGPATFPLSQVIPGWTEGVQLLKEGGEATFYIPSNLAYREQGAGEKIG 239

717 IDGTVFDSSKANGGPATFPLSQVIPGWTEGVQLLKEGGEATFYIPSNLAYREQGAGEKIG 238

229 IDGTVFDSSKANGGPATFPLSQVIPGWTEGVQLLKEGGEATFYIPSNLAYREQGAGEKIG 238

324 IDGTVFDSSKANGGPATFPLSQVIPGWTEGVQLLKEGGEATFYIPSNLAYREQGAGDKIG 239

262 IDGTVFDSSKANGGPATFPLSQVIPGWTEGVQLLKEGGEATFYIPSNLAYREQGAGDKIG 238

9 IDGTVFDSSKANGGPATFPLSQVIPGWTEGVQLLKEGGEATFYIPSNLAYREQGAGEKIG 239

295 IDGTVFDSSKANGGPVTFPLSQVIPGWTEGVQLLKEGGEATFYIPSNLAYREQGAGDKIG 234

130 IDGTVFDSSKANGGPATFPLSQVIPGWTEGVQLLKEGGEATFYIPSNLAYREQGAGDKIG 234

658 IDGTVFDSSKANGGPATFPLSQVIPGWTEGVQLLKEGGEATFYIPSNLAYREQGAGEKIG 239

58 IDGTVFDSSKANGGPATFPLSQVIPGWTEGVQLLKEGGEATFYIPSNLAYREQGAGEKIG 239

374 IDGTVFDSSKANGGPATFPLSQVIPGWTEGVQLLKEGGEATFYIPSNLAYREQGAGDKIG 238

500 IDGTVFDSSKANGGTVTFPLSQVIPGWTEGVQLLKEGGEATFYIPSNLAYREQDAGDKIG 238

10 IDGTVFDSSKANGGPATFPLSQVIPGWTEGVRLLKEGGEATFYIPSNLAYREQGAGEKIG 238

35 IDGTVFDSSKANGGPATFPLSQVIPGWTEGVRLLKEGGEATFYIPSNLAYREQGAGEKIG 238

141 IDGTVFDSSKANGGTVTFPLSQVIPGWTEGVQLLKEGGEATFYIPSNLAYREQGAGEKIG 238

628 IDGTVFDSSKANGGPVTFPLSQVIPGWTEGVQLLKEGGEATFYIPPNLAYREQGAGDKIG 238

435 IDGTVFDSSKANGGPVTFPLSQVIPGWTEGVQLLKEGGEATFYIPSNLAYREQGAGDKIG 238

132 IDGTVFDSSKANGGTVTFPLSQVIPGWTEGVQLLKEGGEATFYIPSNLAYREQGAGDKIG 238

356 IDGTVFDSSKANGGTVTFPLSQVIPGWTEGVQLLKEGGEATFYIPPNLAYREQGAGDKIG 238

6 IDGTVFDSSKANGGPVTFPLSQVIPGWTEGVQLLKEGGEATFYIPSNLAYREQGAGDKIG 238

322 IDGTVFDSSKANGGPVTFPLSQVIPGWTEGVQLLKEGGEATFYIPSNLAYREQGAGDKIG 238

301 IDGTVFDSSKANGGPVTFPLSQVIPGWTEGVQLLKEGGEATFYIPSNLAYREQGAGDKIG 238

630 IDGTVFDSSKANGGPVTFPLSQVIPGWTEGVQLLKEGGEATFYIPSNLAYREQGAGDKIG 238

625 IDGTVFDSSKANGGPVTFPLSQVIPGWTEGVQLLKEGGEATFYIPSNLAYREQGAGDKIG 238

179 IDGTVFDSSKANGGPVTFPLSQVIPGWTEGVQLLKEGGEATFYIPSNLAYREQGAGDKIG 238

63 IDGTVFDSSKANGGPVTFPLSQVIPGWTEGVQLLKEGGEATFYIPPNLAYREQGAGDKIG 238

535 IDGTVFDSSKANGGPVTFPLSQVIPGWTEGVQLLKEGGEATFYIPSNLAYREQGAGDKIG 238

125 IDGTVFDSSKANGGPVTFPLSQVIPGWTEGVQLLKEGGEATFYIPSNLAYREQGAGDKIG 238

217 IDGTVFDSSKANGGPVTFPLSQVIPGWTEGVQLLKEGGEATFYIPSNLAYREQGAGDKIG 238

465 IDGTVFDSSKANGGPVTFPLSQVIPGWTEGVQLLKEGGEATFYIPSNLAYREQGAGDKIG 238

66 IDGTVFDSSKANGGPVTFPLSQVIPGWTEGVQLLKEGGEATFYIPSNLAYREQGAGDKIG 238

3 IDGTVFDSSKANGGPVTFPLSQVILGWTEGVQLLKEGGEATFYIPSNLAYREQGAGDKIG 238

11 IDGTVFDSSKANGGPVTFPLSQVILGWTEGVQLLKEGGEATFYIPSNLAYREQGAGDKIG 238

453 IDGTVFDSSKANGGPVTFPLSQVIPGWTEGVQLLKEGGEATFYIPSNLAYREQGAGDKIG 238

5 IDGTVFDSSKANGGPVTFPLSQVIPGWTEGVQLLKEGGEATFYIPSNLAYREQGAGDKIG 238

727 IDGTVFDSSKANGGPVTFPLSQVIPGWTEGVQLLKESGEATFYIPSNLAYREQGAGDKIG 238

686 IDGTVFDSSKANSGPVTFPLSQVIPGWTEGVQLLKEGGEATFYIPSNLAYREQGAGDKIG 238

608 IDGTVFDSSKANGGPVTFPLSQVIPGWTEGVRLLKEGGEATFYIPSNLAYREQGAGDKIG 238

571 IDGTVFDSSKVNGGPVTFPLSQVIPGWTEGVQLLKEGGEATFYIPSNLAYREQGAGDKIG 238

554 IDGTVFDSSKANGGPVTFPLSQVIPGWIEGVQLLKEGGEATFYIPSNLAYREQGAGDKIG 238

521 IDGTVFDSSKANGGPVTFPLSQVIPGWTEGVQLLKEGGEATFYIPSNLAYREQGAGDKIG 238

517 IDGTVFDSSKANGGPVTFPLSQVIPGWTEGVQLLKEGGEATFYIPSNLAYREQGAGDKIG 238

507 IDGTVFDSSKANGGPVTFPLSQVIPGWTEGVQLLKEGGEATFYIPSNLAYREQGAGDKIG 238

498 IDGTVFDSSKANGGPVTFPLSQVIPGWTEGVQLLKEGGEATFYIPSNLAYREQGAGDKIG 238

486 IDGTVFDSSKANGGPVTFPLSQVIPGWTEGVQLLKEGGEATFYIPSNLAYREQGAGDKIG 238

480 IDGTVFDSSKANGGPVTFPLSQVIPGWTEGVQLLKEGGEATFYIPSNLAYREQGAGDKIG 238

466 IDDTVFDSSKANGGPVTFPLSQVIPGWTEGVQLLKEGGEATFYIPSNLAYREQGAGDKIG 238

440 IDGTVFDSSKANGGPVTFPLSQVIPGWTEGVQLLKEGGEATFYIPSNLAYREQGAGDKIG 238

439 IDGTVFDSSKANGGPVTFPLSQVIPGWTEGVQLLKEGGEATFYIPSNLAYREQGAGDKIG 238

370 IDGTVFDSSKANGGPVTFPLSQVIPGWTEGVQLLKEGGEATFYIPSNLAYREQGAGDKIG 238

367 IDGTVFDSSKANGGPVTFPLSQVIPGWTEGVQLLKEGGEATFYIPSNLAYREQGAGDKIG 238

219 IDGTVFDSSKANGGPVTFPLSQVIPGWTEGVQLLKEGGEATFYIPSNLAYREQGAGDKIG 238

197 IDGTVFDSSKANGGPVTFPLSQVIPGWTEGVQLLKEGGEATFYIPSNLAYREQGAGDKIG 238

494 IDGTVFDSSKANGGPITFPLSQVIPGWTEGVQLLKEGGEATFYIPSNLAYREQGAGDKIG 238

196 IDGTVFDSSKANGGPATFPLSQVIPGWTEGVQLLKEGGEATFYIPSNLAYREQGAGDKIG 238

182 IDGTVFDSSKANGGPVTFPLSQVIPGWTEGVQLLKEGGEATFYIPSNLAYREQGAGDKIG 238

265 IEGTVFDSSKANGGPVTFPLSQVIPGWTEGVQLLKEGGEATFYIPSNLAYREQGAGDKIG 238

155 IGGTVFDSSKANGGPVTFPLSQVIPGWTEGVQLLKEGGEATFYIPSNLAYREQGAGDKIG 238

114 IDGTVFDSSKANGGPVTFPLSQVIPGWTEGVQLLKEGGEATFYIPSNLAYREQGAGDKIG 238

112 IDGTVFDSSKANGGPVTFPLSQVIPGWTEGVQLLKEGGEATFYIPSNLAYREQGAGDKIG 238

110 IDGTVFDSSKANGGPVTFPLSQVIPGWTEGVQLLKEGGEATFYIPSNLAYREQGAGDKIG 238

74 IDGTVFDSSKANGGPVTFPLSQVIPGWTEGVQLLKEGGEATFYIPSNLAYREQGAGDKIG 238

659 IDGTVFDSSKANGGPVTFPLSQVIPGWTESVQLLKEGGEATFYIPSNLAYREQGAGDKIG 238

627 IDGTVFDSSKANGGPVTFPLSQVIPGWTEGVQLLKEGGEATFYIPSNLAYREQGAGDKIG 238

502 IDGTVFDSSKANGGPVTFPLSQVIPGWTEGVQLLKEGGEATFYIPSNLAYREQGAGDKIG 238

371 IDGTVFDSSKANGGPVTFPLSQVIPGWTEGVQLLKEGGEATFYIPSNLAYREQGAGDKIG 238

320 IDGTVFDSSKANGGPVTFPLSQVIPGWTEGVQLLKEGGEATFYIPSNLAYREQGAGDKIG 238

290 IDGTVFDSSKANGGPVTFPLSQVIPGWTEGVQLLKEGGEATFYIPSNLAYREQGAGDKIG 238

216 IDGTVFDSSKANGGPVTFPLSQVIPGWTEGVQLLKEGGEATFYIPSNLAYREQGAGEKIG 238

146 IDGTVFDSSKANGGPVTFPLSQVIPGWTEGVQLLKEGGEATFYIPSNLAYREQGSGDKIG 238

109 IDGTVFDSSKANGGPVTFPLSQVIPGWTEGVQLLKEGGEATFYIPSNLAYREQGAGDKIG 238

572 IDGTVFDSSKANGGPVTFPLSQVIPGWTEGVQLLKEGGEATFYIPSNLAYREQGAGDKIG 238

540 IDGTVFDSSKANGGPVTFPLSQVIPGWTEGVQLLKEGGEATFYIPSNLAYREQGAGDKIG 238

365 IDGTVFDSSKANGGPVTFPLSQVIPGWTEGVQLLKEGGEATFYIPSNLAYREQGAGDKIG 238

148 IDGTVFDSSKANGGPVTFPLSQVIPGWTEGVQLLKEGGEATFYIPSNLAYREQGAGDKIG 238

726 IDGTVFDSSKANGGPVTFPLSQVIPGWTEGVQLLKEGGEATFYIPSNLAYREQGAGDKIG 238

729 IDGTVFDSSKANGGPVTFPLSQVIPGWTEGVQLLKEGGEATFYIPSNLAYREQGAGDKIG 238

331 IDGTVFDSSKANGGPVTFPLSQVIPGWTEGVQLLKEGGEATFYIPSNLAYREQGAGDKIG 238

396 IDGTVFDSSKANGGPVTFPLSQVIPGWTEGVQLLKEGGEATFYIPSNLAYREQGAGDKIG 238

406 IDGTVFDSSKANGGPVTFPLSQVIPGWTEGVQLLKEGGEATFYIPSNLAYREQGAGDKIG 238

438 IDGTVFDSSKANGGPVTFPLSQVIPGWTEGVQLLKEGGEATFYIPSNLAYREQGAGDKIG 238

483 IDGTVFDSSKANGGPVTFPLSQVIPGWTEGVQLLKEGGEATFYIPSNLAYREQGAGDKIG 238

570 IDGTVFDSSKANGGPVTFPLSQVIPGWTEGVQLLKEGGEATFYIPSNLAYREQGAGDKIG 238

655 IDGTVFDSSKANGGPVTFPLSQVIPGWTEGVQLLKEGGEATFYIPSNLAYREQGAGDKIG 238

716 IDGTVFDSSKANGGPVTFPLSQVIPGWTEGVQLLKEGGEATFYIPSNLAYREQGAGDKIG 238

147 IDGTVFDSSKANGGPVTFPLSQVIPGWTEGVQLLKEGGGATFYIPSNLAYREQGAGDKIG 238

321 IDGTVFDSSKANGGPVTFPLSQVIPGWTEGVQLLKEGGEATFYIPSNLAYREQGAGDKIG 238

688 IDGTVFDSSKANGGPVTLPLSQVIPGWTEGVQLLKEGGEATFYIPSNLAYREQGAGDKIG 238

299 IDGTVFDSGKANGGPVTFPLSQVIPGWTEGVQLLKEGGEATFYIPSNLAYREQGAGDKIG 238

622 IDGTVFDSSKANGGPVTFPLSQVIPGWTEGVQLLKEGGEATFYIPSNLAYREQGAGDKIG 238

405 IDGTVFDSSKANGGPVTFPLSQVIPGWTEGVQLLKEGGEATFYIPSNLAYREQGAGDKIG 238

528 IDGTVFDSSKANGGPVTFPLSQVIPGWTEGVQLLKEGGEATFYIPSNLAYREQGAGDKIG 238

94 IDGTVFDSSKANGGPVTFPLSQVIPGWTEGVQLLKEGGEATFYIPSNLAYREQGAGDKIG 238

399 IDGTVFDSSKANGGPVTFPLSQVIPGWTEGVQLLKEGGEATFYIPSNLAYREQGAGDKIG 238

2 IDGTVFDSSKANGGPVTFPLSQVIPGWTEGVQLLKEGGEATFYIPSNLAYREQGAGDKIG 238

152 IDGTVFDSSKANGGTVTFPLSQVIPGWTEGVQLLKEGGEATFYIPSNLAYREQGAGDKIG 238

415 IDGTVFDSSKANGGPVTFPLSQVIPGWTEGVQLLKEGGEATFYIPSNLAYREQGAGDKIG 238

323 IDGTVFDSSKANGGTVTFPLSQVIPGWTEGVQLLKEGGEATFYIPSNLAYREQGAGDKIS 238

96 IDGTVFDSSKANGGTVTFPLSQVIPGWTEGVQLLKEGGEATFYIPSNLAYREQGAGDKIG 238

529 IDGTVFDSSKANGGPVTFPLSQVIPGWTEGVQLLKEGGEATFYIPSNLAYREQGAGEKIG 238

541 IDGTVFDSSKANGGPATFPLSQVIPGWTEGVQLLKEGGEATFYIPSNLAYREQGAGEKIG 238

687 IDGTVFDSSKANGGPATFPLSQVIPGWTEGVQLLKEGGEATFYIPSNLAYREQGAGEKIG 238

107 IDGTVFDSSKANGGPVTFPLSQVIPGWTEGVQLLKEGGEATFYIPSNLAYREQGAGDKIG 234

266 IDGTVFDSSKANGGPVTFPLSQVIPGWTEGVQLLKEGGEATFYIPSNLAYREQGAGDKIG 234

545 IDGTVFDSSKANGGPVTFPLSQVIPGWTEGVQLLKEGGEATFYIPSNLAYREQGAGDKIG 234

685 IDGTVFDSSKANGGPVTFPLSQVIPGWTEGVQLLKEGGEATFYIPSNLAYREQGAGDKIG 234

7 IDGTVFDSSKANGGPVTFPLSQVIPGWTEGVQLLKEGGEATFYIPSNLAYREQGAGDKIG 234

549 IDGTVFDSSKANGGPVTFPLSQVIPGWTEGVQLLKEGGEATFYIPSNLAYREQGAGDKIG 234

553 IDGTVFDSSKANGGPVTFPLSQVIPGWTEGVQLLKEGGEATFYIPSNLAYREQGAGDKIG 234

222 IDGTVFDSSKANGGPVTFPLSQVIPGWTEGVQLLKEGGEATFYIPSNLAYREQGAGDKIG 234

157 IDGTVFDSSKANGGPVTFPLSQVIPGWTEGVQLLKEGGEATFYIPSNLAYREQGAGDKIG 234

294 IDGTVFDSSKANGGPVTFPLSQMIPGWTEGVQLLKEGGEATFYIPSNLAYREQGAGDKIG 234

441 IDGTVFDSSKANGGPVTFPLSQVIPGWTEGVQLLKEGGEATFYIPSNLAYREQGAGDKIG 234

654 IDGTVFDSSKANGGPVTFPLSQVIPGWTEGVQLLKEGGEATFYIPSNLAYREQGAGDKIG 234

736 IDGTVFDSSKANGGPVTFPLSQVIPGWTEGVQLLKEGGEATFYIPSNLAYREQGAGDKIG 234

240 IDGTVFDSSKANGGPVTFPLSQVILGWTEGVQLLKEGGEATFYIPSNLAYREQGAGDKIG 234

22 IDGTVFDSSKANGGPVTFPLSQVIPGWTEGVQLLKEGGEATFYIPSNLAYREQGAGDKIG 234

188 IDGTVFDSSKANGGPVTFPLSQVIPGWTEGVQLLKEGGEATFYIPSNLAYREQGAGDKIG 234

638 IDGTVFDSSKANGGPVTFPLSQVIPGWTEGVQLLKEGGEATFYIPSNLAYREQGAGDKIG 234

106 IDGTVFDSSKANGGPVTFPLSQVIPGWTEGVQLLKEGGEATFYIPSNLAYREQGAGDKIG 234

105 IDGTVFDSSKANGGPVTFPLSQVIPGWTEGVQLLKEGGEATFYIPSNLAYREQGAGDKIG 234

166 IDGTVFDSSKANGGPVTFPLSQVIPGWTEGVQLLKEGGEATFYIPSNLAYREQGAGDKIG 234

189 IDGTVFDSSKANGGPVTFPLSQVIPGWTEGVQLLKEGGEATFYIPSNLAYREQGAGDKIG 234

215 IDGTVFDSSKANGGPVTFPLSQVIPGWTEGVQFLKEGGEATFYIPSNLAYREQGAGDKIG 234

259 IDGTVFDSSKANGGPVTFPLSQVIPGWTEGVQLLKEGGEATFYIPSNLAYREQGAGDKIG 234

287 IDGTVFDSSKANGGPVTFPLSQVIPGWTEGVQLLKEGGEATFYIPSNLTYREQGAGDKIG 234

366 IDGTVFDSSKANGGPVTFPLSQVIPGWTEGVQLLKEGGEATFYIPSNLAYREQGAGDKIG 234

419 IDGTVFDSSKANGGPVTFPLSQVIPGWTEGVQLLKEGGEATFYIPSNLAYREQGAGDKIG 234

490 IDGTVFDSSKANGGSVTFPLSQVIPGWTEGVQLLKEGGEATFYIPSNLAYREQGAGDKIG 234

589 IDGTVFDSSKANGGPVTFPLSQVIPGWTEGVQLLKEGGEATFYIPSNLAYREQGAGDKIG 234

657 IDGTVFDSSKANGGPVTFPLSQVIPGWTEGVQLLKEGGEATFYIPSNLAYREQGAGDKIG 234

680 IDGTVFDSSKANGGPVTFPLSQVIPGWTEGVQLLKEGGEATFYIPSNLAYREQGAGDKIG 234

526 IDGTVFDSSKANGGPVTFPLSQVIPGWTEGVQLLKEGGEATFYIPSNLAYREQGAGDKIG 234

708 IDGTVFDSSKANGGPVTFPLSQVIPGWTEGVQLLKEGGEATFYIPSNLAYREQGAGDKIG 234

710 IDGTVFDSSKANGGPVTFLLSQVIPGWTEGVQLLKEGGEATFYIPSNLAYREQGAGDKIG 234

1 IDGTVFDSSKANGGPVTFPLSQVIPGWTEGVQLLKEGGEATFYIPSNLAYREQGAGDKIG 234

512 IDGTVFDSSKANGGPVTFPLSQVIPGWTEGVQLLKEGGEATFYIPSNLAYREQGAGDKID 238

604 IDGTVFDSSKANGGPVTFPLSQVIPGWTEGVQLLKEGGEATFYIPSNLAYREQGAGDKIG 238

24 IDGTVFDSSKANGGPVTFPLSQVIPGWTEGVQLLKEGGEATFYIPSNLAYREQGAGDKIG 238

116 IDGTVFDSSKANGGPVTFPLSQVIPGWTEGVQLLKEGGEATFYIPSNLAYREQGAGDKIG 238

359 IDGTVFDSSKANGGPVTFPLSQVIPGWTEGVQLLKEGGEATFYIPSNLAYREQGAGDKIG 238

653 IDGTVFDSSKANGGTVTFPLSQVIPGWTEGVQLLKEGGEATFYIPSNLAYREQGAGDKIG 238

568 IDGTVFDSSKANGGTVTFPLSQVIPGWTEGVQLLKEGGEATFYIPSNLAYREQGAGDKIG 238

13 IDGTVFDSSKANGGTVTFPLSQVIPGWTEGVQLLKEGGEATFYIPSNLAYREQGAGDKIG 238

257 IDGTVFDSSKANGGTVTFPLSQVIPGWTEGVQLLKEGGEATFYIPSNLAYREQGAGDKIG 238

* .*****.*.*.* *: ***:* ** *.*::***.* ****** **:****.:*:**.

15 PNATLVFDVKLVKVGAPENASAQQPVQVDVKKVN 273

319 PNSTLVFDVKLVKVGAPGNASAQQPAQVDIKKVN 268

27 PNSTLVFDVKLVKVGAPENASAQQPAQVDIKKVN 274

88 PNSTLVFDVKLVKVGAPENASAQQPAQVDIKKVN 272

113 PNSTLVFDVKLVKVGAPGNASAQQPAQVDIKKVN 273

717 PNATLVFDVKLVKVGAPENASAQQPAQVNIKKVN 272

229 PNSTLVFDVKLVKVGAPENASAQQPAQVDIKKVN 272

324 PNSTLVFDVKLVKVGAPENAPAQQPVQVDVKKVN 273

262 PNATLVFDVKLVKVGAPENAPAQQPVQVDVKKVN 272

9 PNATLVFDVKLVKVGAPENAPAKQPVQVDIKKVN 273

295 PNATLVFDVKLVKVGAPENAPTQQPVQVDVKKVN 268

130 PNATLVFDVKLVKVGAPENAPAKQPVQVDIKKVN 268

658 PNATLVFDVKLVKVGAPENAPAKQPVQVDIKKVN 273

58 PNATLVFDVKLVKVGAPENAPAKQPVQVDIKKVN 273

374 PNATLVFDAKLVKVGAPENAPAKQPVQVDIKKVN 272

500 PNATLVFDVKLVKIGAPENAPAKQPAQVDIKKVN 272

10 PNATLVFDVKLVKIGAPENAPAKQPDQVDIKKVN 272

35 PNATLVFDVKLVKIGAPENAPAKQPDQVDIKKVN 272

141 PNATLVFDVKLVKVGAPENAPAQQPVQVDVKKVN 272

628 PNATLVFDVKLVKIGAPENAPAKQPVQVDIKKVN 272

435 PNATLVFDVKLVKIGAPENAPAKQPAQVDIKKVN 272

132 PNATLVFDVKLVKIGAPENAPAKQPAQVDIKKVN 272

356 PNATLVFDVKLVKIGAPENAPAKQPAQVDIKKVN 272

6 PNATLVFDVKLVKIGAPENAPAKQPAQVDIKKVN 272

322 PNATLVFDVKLVKIGAPENAPAKQPAQVDIKKVN 272

301 PNATLVFDVKLVKIGAPENASAKQPAQVDIKKVN 272

630 PNATLVFDVKLVKIGAPENAPAKQPAQVDIKKVN 272

625 PNATLVFDVKLVKIGAPENAPAKQPAQVDIKKVN 272

179 PNATLVFDVKLVKIGAPENAPAKQPAQVDIKKVN 272

63 PNATLVFDVKLVKIGAPENAPAKQPAQVDIKKVN 272

535 PNTTLVFDVKLVKIGAPENAPAKQPAQVDIKKVN 272

125 PNTTLVFDVKLVKIGAPENAPAKQPAQVDIKKVN 272

217 PNATLVFDVKLVKIGAPENAPAKQSAQVDIKKVN 272

465 PNATLVFDVKLVKIGAPENAPAKQPAQVDIKKVN 272

66 PNATLVFDVKLVKIGAPENAPAKQPAQVDIKKVN 272

3 PNATLVFDVKLVKIGAPENAPAKQPAQVDIKKVN 272

11 PNATLVFDVKLVKIGAPENAPAKQPAQVDIKKVN 272

453 PNATLVFDVKLVKIGAPENAPAKQPAQVDIKKVN 272

5 PNATLVFDVKLVKIGAPENAPAKQPAQVDIKKVN 272

727 PNATLVFDVKLVKIGAPENAPAKQPAQVDIKKVN 272

686 PNATLVFDVKLVKIGAPENAPAKQPAQVDIKKVN 272

608 PNATLVFDVKLVKIGAPENAPAKQPAQVDIKKVN 272

571 PNATLVFDVKLVKIGAPENAPAKQPAQVDIKKVN 272

554 PNATLVFDVKLVKIGAPENAPAKQPAQVDIKKVN 272

521 PNATLVFDVKLVKIGAPENAPAKQPAQVDIKKVN 272

517 PNATLVFDVKLVKIGAPENAPAKQPAQVDIKKVN 272

507 PNATLVFDVKLVKIGAPENAPVKQPAQVDIKKVN 272

498 PNATLVFDVKLVKIGAPENAPAKQPAQVDIKKVN 272

486 PNATLVFDVKLVKIGAPENAPAKQPAQVDIKKVN 272

480 PNATLVFDVKLVKIGAPENAPAKQPAQVDIKKVN 272

466 PNATLVFDVKLVKIGAPENAPAKQPAQVDIKKVN 272

440 PNATLVFDVKLVKIGAPENAPAKQPAQVDIKKVN 272

439 PNATLVFDVKLVKIGAPENAPAKQPAQVDIKKVN 272

370 PNATLVFDVKLVKIGAPENAPAKQPAQVDIKKVN 272

367 PNATLVFDVKLVKIGAPENAPAKQPAQVDIKKVN 272

219 PNATLVFDVKLVKIGAPENAPAKQPAQVDIKKVN 272

197 PNATLVFDVKLVKIGAPENAPAKQPAQVDIKKVN 272

494 PNATLVFDVKLVKIGAPENAPAKQPAQVDIKKVN 272

196 PNATLVFDVKLVKIGAPENAPAKQPAQVDIKKVN 272

182 PNATLVFDVKLVKIGALENAPAKQPAQVDIKKVN 272

265 PNATLVFDVKLVKIGAPENAPAKQPAQVDIKKVN 272

155 PNATLVFDVKLVKIGAPENAPAKQPAQVDIKKVN 272

114 PNATLVFDVKLVKIGAPENAPAKQPAQVDIKKVN 272

112 PNATLVFDVKLVKIGAPENAPAKQPAQVDIKKVN 272

110 PNATLVFDVKLVKIGAPENAPAKQPAQVDIKKVN 272

74 PNATLVFDVKLVKIGAPENAPAKQPAQVDIKKVN 272

659 PNATLVFDVKLVKIGAPENAPAKQPAQVDIKKVN 272

627 PNATLVFDVKLVKIGAPENAPAKQPAQVDIKKVN 272

502 PNATLVFDVKLVKIGAPENAPAKQPAQVDIKKVN 272

371 PNATLVFDVKLVKIGAPENATAKQPAQVDIKKVN 272

320 PNATLVFDVKLVKIGAPENAPAKQPAQVDIKKVN 272

290 PNATLVFDVKLVKIGAPENAPAKQPAQVDIKKVN 272

216 PNATLVFDVKLVKIGAPENAPAKQPAQVDIKKVN 272

146 PNATLVFDVKLVKIGAPENAPAKQPAQVDIKKVN 272

109 PNATLVFDVKLVKIGAPENAPAKQPAQVDIKKVN 272

572 PNATLVFDVKLVKIGAPENAPAKQSAQVDIKKVN 272

540 PNATLVFDVKLVKIGAPENAPAKQPAQVDIKKVN 272

365 PNATLVFDVKLVKIGAPENAPAKQPAQVDIKKVN 272

148 PNATLVFDVKLVKIGAPENAPAKQPVQVDIKKVN 272

726 PNATLVFDVKLVKIGAPENAPAKQPAQVDIKKVN 272

729 PNATLVFDVKLVKIGAPENAPAKQPAQVDIKKVN 272

331 PNATLVFDVKLVKIGAPENAPAKQPAQVDIKKVN 272

396 PNATLVFDVKLVKIGAPENAPAKQPAQVDIKKVN 272

406 PNATLVFDVKLVKIGAPENAPAKQPAQVDIKKVN 272

438 PNATLVFDVKLVKIGAPENAPAKQPAQVDIKKVN 272

483 PNATLVFDVKLVKIGAPENAPAKQPAQVDIKKVN 272

570 PNATLVFDVKLVKIGAPENAPAKQPAQVDIKKVN 272

655 PNATLVFDVKLVKIGAPENAPAKQPAQVDIKKVN 272

716 PNATLVFDVKLVKIGAPENAPAKQPAQVDIKKVN 272

147 PNATLVFDVKLVKIGAPENAPAKQPAQVDIKKVN 272

321 PNATLVFDVKLVKIGAPENAPAKQPAQVDIKKVN 272

688 PNATLVFDVKLVKIGAPENAPAKQPAQVDIKKVN 272

299 PNATLVFDVKLVKIGAPENAPAKQPAQVDIKKVN 272

622 PNATLVFDVKLVKIGAPENAPAKQPAQVDIKKVN 272

405 PNATLVFDVKLVKIGAPENAPAKQPAQVDIKKVN 272

528 PNATLVFDVKLVKIGAPENAPAKQPAQVDIKKVN 272

94 PNATLVFDVKLVKIGAPENAPAKQPAQVDIKKVN 272

399 PNATLVFDVKLVKIGAPENAPAKQPAQVDIKKVN 272

2 PNATLVFDVKLVKIGAPENAPAKQPAQVDIKKVN 272

152 PNATLVFDVKLVKIGAPENAPAKQPAQVDIKKVN 272

415 PNATLVFDVKLVKIGAPENAPAKQPAQVDIKKVN 272

323 PNATLVFDVKLVKIGAPENAPAKQPAQVDIKKVN 272

96 PNATLVFDVKLVKIGAPENAPAKQPAQVDIKKVN 272

529 PNSTLVFDVKLVKVGAPGNASAQQPAQVDIKKVN 272

541 PNATLVFDVKLVKVGAPENAPAKQPVQVDIKKVN 272

687 PNATLVFDVKLVKVGAPENAPAKQPVQVDIKKVN 272

107 PNATLVFDVKLVKIGAPENAPAKQPAQVDIKKVN 268

266 PNATLVFDVKLVKIGAPENAPAKQPAQVDIKKVN 268

545 PNATLVFDVKLVKIGAPENAPAKQPDQVDIKKVN 268

685 PNTTLVFDVKLVKIGAPENAPAKQPAQVDIKKVN 268

7 PNATLVFDVKLVKIGAPENAPAKQPAQVDIKKVN 268

549 PNATLVFDVKLVKVGAPENAPAKQPVQVDIKKVN 268

553 PNATLVFSVKLVNIGAPENAPAKQPAQVDIKKVN 268

222 PNATLVFDVKLVKIGAPENAPAKQPAQVDIKKVN 268

157 PNATLVFDVKLVKIGAPENAPAKQPAQVDIKKVN 268

294 PNATLVFDVKLVKIGAPENAPAKQPAQVDIKKVN 268

441 PNATLVFDVKLVKIGAPENAPAKQPAQVDIKKVN 268

654 PNATLVFDVKLVKIGAPENAPTKQPAQVDIKKVN 268

736 PNATLVFDVKLVKIGAPENAPAKQPAQVDIKKVN 268

240 PNATLVFDVKLVKIGAPENAPAKQPAQVDIKKVN 268

22 PNATLVFDVKLVKIGAPENAPAKQPAQVDIKKVN 268

188 PNATLVFDVKLVKIGAPENAPAKQPAQVDIKKVN 268

638 PNATLVFDVKLVKIGAPENAPAKQSAQVDIKKVN 268

106 PNATLVFDVKLVKIGSPENAPAKQPAQVDIKKVN 268

105 PNATLVFDVKLVKIGAPENAPAKQPAQVDIKKVN 268

166 PNATLVFDVKLVKIGAPENAPAKQPAQVDIKKVN 268

189 PNATLVFDVKLVKIGAPENAPAKQPAQVDIKKVN 268

215 PNATLVFDVKLVKIGAPENAPAKQPAQVDIKKVN 268

259 PNATLVFDVKLVKIGAPENAPAKQPAQVDIKKVN 268

287 PNATLVFDVKLVKIGAPENAPAKQPAQVDIKKVN 268

366 PNATLVFDVKLVKIGAPENVPAKQPAQVDIKKVN 268

419 PNATLVFDVKLVKIGAPENAPAKQPAQVDIKKVN 268

490 PNATLVFDVKLVKIGAPENAPAKQPAQVDIKKVN 268

589 PNATLVFDVKLVKIGALENAPAKQPAQVDIKKVN 268

657 PNATLVFDVKLVKIGAPENAPAKQPVQVDIKKVN 268

680 PNATLVFDVKLVKIGAPENAPAKQPAQVDIKKVN 268

526 PNATLVFDVKLVKIGAPENAPAKQPAQVDIKKVN 268

708 PNATLVFDVKLVKIGAPENAPAKQPAQVDIKKVN 268

710 PNATLVFDVKLVKIGAPENAPAKQPAQVDIKKVN 268

1 PNATLVFDVKLVKIGAPENAPAKQPAQVDIKKVN 268

512 PNATLVFDVKLVKIGAPENAPAKQPAQVDIKKVN 272

604 PNATLVFDVKLVKIGAPENAPAKQPAQVDIKKVN 272

24 PNATLVFDVKLVKIGAPENAPAKQPAQVDIKKVN 272

116 PNATLVFDVKLVKVGAPENAPTQQPVQVDVKKVN 272

359 PNATLVFDVKLVKIGAPENAPAKQPAQVDIKKVN 272

653 PNATLVFDVKLVKIGAPENAPAKQPAQVDIKKVN 272

568 PNATLVFDVKLVKIGAPENAPAKQPAQVDIKKVN 272

13 PNATLVFDVKLVKIGAPENAPAKQPAQVDIKKVN 272

257 PNATLVFDVKLVKIGAPENAPSKQPAQVDIKKVN 272

**:****..***::*: *. :* **::****
