# Supplementary material for: Update on the Neisseria Macrophage Infectivity Potentiator-Like PPIase Protein
Source: Front Cell Infect Microbiol. 2022 Mar 22;12:861489. doi: 10.3389/fcimb.2022.861489 (PMC8981591; doi:10.3389/fcimb.2022.861489)
Supplement: Supplementary file 8 [file Table_5.docx]

**Supplementary Dataset 5. A Clustal alignment of MIP sequences from gonococci, meningococci and several commensal species.**

19091_oralis MNKTFKLSALALAAALTVTACNKKDAAAPAASA----PAASGAEASAIGTPAQQASYAMG 56

3565_mucosa MNKTFKFSALAIAAALALTACDKKDANTPAAASAPASAASAATDSSSIGTPTQQASYAMG 60

43658_subflava MNKTFKFSALALSALLALTACNQKDTAAKDAP-AASAASNATADASAIGSTAQQASYAMG 59

38978_cinerea MNKIFKISALTLAATLALSACGKKETVPTSSASEPAAASAAQGDTSSIGNTMQQASYAMG 60

140_Ng MNTIFKISALTLSAALALSACGKKEAAPAS-ASEPAAASAAQGDTSSIGSTMQQASYAMG 59

10_Ng MNTIFKISALTLSAALALSACGKKEAAPAS-ASEPAAASAAQGDTSSIGGTMQQASYAMG 59

35_Ng MNTIFKISALTLSAALALSACGKKEAAPAS-ASEPAAASAAQGDTSSIGGTMQQASYAMG 59

2_Nm MNTIFKISALTLSAALALSACGKKEAAPAS-ASEPAAASSAQGDTSSIGSTMQQASYAMG 59

13_Nm MNTIFKISALTLSAALALSACGKKEAAPAS-ASEPAAASSAQGDTSSIGSTMQQASYAMG 59

7_Nm MNTIFKISALTLSAALALSACGKKEAA-----SEPAAASAAQGDTSSIGSTMQQASYAMG 55

1_Nm MNTIFKISALTLSAALALSACGKKEAA-----SEPAAASAAQGDTSSIGSTMQQASYAMG 55

22_Nm MNTIFKISALTLSAALALSACGKKEAA-----SEPAAASAAQGDTSSIGSTMQQASYAMG 55

44518_benedictiae MNKIFKISALTLAATLALSACGKKENVPASSASEPAAASAAQGDTSSIGNTMQQASYAMG 60

1770_lactamica MNTIFKISALTLSAALALSACGKKEAAPAATASEPAAASAAQGDTSSIGSTMQQASYAMG 60

14730_polysaccharea MNKIFKISALTLAATLALSACGKKEAAPA-SASEPAAASAAQGDTSSIGSTMQQASYAMG 59

46231_blantyrii MNKIFKISALTLAATLALSACGKKEAAPA-SASEPAAASAAQGDTSSIGSTMQQASYAMG 59

43425_maigaei MNTIFKISALTLSAALALSACGKKENVPA-SASEPAAASAAQGDTSSIGSTMQQASYAMG 59

46405_viridae MNTIFKISALTLSAALALSACGKKEAAPA-SASEPAAASAAQGDTSSIGSTMQQASYAMG 59

46192_basseii MNTIFKISALTLSAALALSACGKKEAAPA-SASEPAAASAAQGDTSSIGSTMQQASYAMG 59

42909_bergeri MNTIFKISALTLSAALALSACGKKEAAPA-SASEPAAASAAQGDTSSIGSTMQQASYAMG 59

43128_uirgultaei MNTIFKISALTLSAALALSACGKKEAAPA-SASEPAAASAAQGDTSSIGSTMQQASYAMG 59

**. **:***:::* *:::**.:*: : : ::*:** ********

19091_oralis VDIGRSLKQMKDQGTDIDLKVFNEAIQTMYDGKEPKMNEMQAQEVMLKFLQEQQTKAQAK 116

3565_mucosa MDIGRSLKQMKDQGAEIDMKVFIEAMQASYEGKENKMTEAQAQEVMMKFLTEQQTKAVEK 120

43658_subflava VDIGRSLKQMKDQGTEIDLKVFTEAMEAMFEGKEVKMTEAQAQEVMMKFLQEQQEKATAK 119

38978_cinerea VDIGRSLKQMKEQGAEIDLKVFTEALQAMYDGKEIKMTEEQAQEVMMKFLQEQQAKAVEK 120

140_Ng VDIGRSLKQMKEQGAEIDLKVFTDAMQAVYDGKEIKMTEEQAQEVMMKFLQEQQAKAVEK 119

10_Ng VDIGRSLKQMKEQGAEIDLKVFTDAMQAVYDGKEIKMTEEQAQEVMMKFLQEQQAKAVEK 119

35_Ng VDIGRSLKQMKEQGAEIDLKVFTDAMQAVYDGKEIKMTEEQAQEVMMKFLQEQQAKAVEK 119

2_Nm VDIGRSLKQMKEQGAEIDLKVFTEAMQAVYDGKEIKMTEEQAQEVMMKFLQEQQAKAVEK 119

13_Nm VDIGRSLKQMKEQGAEIDLKVFTEAMQAVYDGKEIKMTEEQAQEVMMKFLQEQQAKAVEK 119

7_Nm VDIGRSLKQMKEQGAEIDLKVFTEAMQAVYDGKEIKMTEEQAQEVMMKFLQEQQAKAVEK 115

1_Nm VDIGRSLKQMKEQGAEIDLKVFTEAMQAVYDGKEIKMTEEQAQEVMMKFLQEQQAKAVEK 115

22_Nm VDIGRSLKQMKEQGAEIDLKVFTEAMQAVYDGKEIKMTEEQAQEVMMKFLQEQQAKAVEK 115

44518_benedictiae VDIGRSLKQMKEQGAEIDLKVFTEAMQAMYDGKEIKMTEEQAQEVMMKFLQEQQAKAVEK 120

1770_lactamica VDIGRSLKQMKEQGAEIDLKVFTEAMQAVYEGKEIKMTEEQAQEVMMKFLQEQQAKAVEK 120

14730_polysaccharea VDIGRSLKQMKEQGAEIDLKVFTEAMQAVYDGKEIKMTEEQAQEVMMKFLQEQQAKAVEK 119

46231_blantyrii VDIGRSLKQMKEQGAEIDLKVFTEAMQAVYDGKEIKMTEEQAQEVMMKFLQEQQAKAVEK 119

43425_maigaei VDIGRSLKQMKEQGAEIDLKVFTEAMQAVYDGKEIKMTEEQAQEVMMKFLQEQQAKAVEK 119

46405_viridae VDIGRSLKQMKEQGAEIDLKVFTEAMQAVYDGKEIKMTEEQAQEVMMKFLQEQQAKAVEK 119

46192_basseii VDIGRSLKQMKEQGAEIDLKVFTEAMQAVYDGKEIKMTEEQAQEVMMKFLQEQQAKAVEK 119

42909_bergeri VDIGRSLKQMKEQGAEIDLKVFTEAMQAVYDGKEIKMTEEQAQEVMMKFLQEQQAKAVEK 119

43128_uirgultaei VDIGRSLKQMKEQGAEIDLKVFTEAMQAVYDGKEIKMTEEQAQEVMMKFLQEQQAKAVEK 119

:**********:**::**:*** :*::: ::*** **.* ******:*** *** ** *

19091_oralis RLEDAKTNLEKGDAFLKENASKEGVKTTASGLQYKVKTEGTGASPKATDIVTVEYEGRLI 176

3565_mucosa LQADAKANLEKGEAFLKENATKEGVKTTASGLQYKITKEGEGKQPTKDDIVVVEYEGRLI 180

43658_subflava RAEDAKVNLEKGEAFLKENATKEGVKTSASGLQYKITKEGEGKKPTKDDMVTVEYEGRLI 179

38978_cinerea HKADAKANKEKGEAFLKENAAKDGVKTTASGLQYKITKQGEGKQPTKDDIVTVEYEGRLI 180

140_Ng HKADAKANKEKGEAFLKENAAKDGVKTTASGLQYKITKQGKGKQPTKDDIVTVEYEGRLI 179

10_Ng HKADAKANKEKGEAFLKENAAKDGVKTTASGLQYKITKQGKGKQPTKDDIVTVEYEGRLI 179

35_Ng HKADAKANKEKGEAFLKENAAKDGVKTTASGLQYKITKQGEGKQPTKDDIVTVEYEGRLI 179

2_Nm HKADAKANKEKGEAFLKENAAKDGVKTTASGLQYKITKQGEGKQPTKDDIVTVEYEGRLI 179

13_Nm HKADAKANKEKGEAFLKENAAKDGVKTTASGLQYKITKQGEGKQPTKDDIVTVEYEGRLI 179

7_Nm HKAEAKANKEKGEAFLKENAAKDGVKTTASGLQYKITKQGEGKQPTKDDIVTVEYEGRLI 175

1_Nm HKADAKANKEKGEAFLKENAAKDGVKTTASGLQYKITKQGEGKQPSKDDIVTVEYEGRLI 175

22_Nm HKADAKANKEKGEAFLKENAAKDGVKTTASGLQYKITKQGEGKQPTKDDIVTVEYEGRLI 175

44518_benedictiae HKADAKANKEKGEAFLKENAAKDGVKTTASGLQYKITKQGEGKQPTKDDIVTVEYEGRLI 180

1770_lactamica HKADAKANKEKGEAFLKENAAKEGVKTTASGLQYKITKQGEGKQPTKDDIVTVEYEGRLI 180

14730_polysaccharea HKADAKANKEKGEAFLKENAAKEGVKTTASGLQYKITKQGEGKQPTKDDIVTVEYEGRLI 179

46231_blantyrii HKADAKANKEKGEAFLKENAGKEGVKTTASGLQYKITKQGEGKQPTKDDIVTVEYEGRLI 179

43425_maigaei HKADAKANKEKGEAFLKENAAKDGVKTTASGLQYKITKQGEGKQPTKDDIVTVEYEGRLI 179

46405_viridae HKADAKANKEKGEAFLKENAGKEGVKTTASGLQYKITKQGEGKQPTKDDIVTVEYEGRLI 179

46192_basseii HKADAKANKEKGEAFLKENAAKEGVKTTASGLQYKITKQGEGKQPTKDDIVTVEYEGRLI 179

42909_bergeri HKADAKANKEKGEAFLKENAGKEGVKTTASGLQYKITKQGEGKQPTKDDIVTVEYEGRLI 179

43128_uirgultaei HKADAKANKEKGEAFLKENAGKEGVKTTASGLQYKITKQGEGKQPTKDDIVTVEYEGRLI 179

:**.* ***:******* *:****:*******:..:* * .*. *:*.********

19091_oralis DGTVFDSSKQNGGQPVTFPVNQVIPGWSEAVQLMKEGGEATFFIPAKLAYGENG-AGDKI 235

3565_mucosa DGTVFDSSKANGS-PATFPVSQVIPGWTEGIQLLKEGGEATFYIPSKLAYRDQAVPGGKI 239

43658_subflava DGTVFDSSKANGG-PVSFPVSQVIPGWTEGIQLLKEGGEATFYIPAKLAYREVG-AGDKI 237

38978_cinerea DGTVFDSSKANGG-PATFPLSQVIPGWTEGVQLLKEGGEATFYIPSNLAYREQG-AGEKI 238

140_Ng DGTVFDSSKANGG-PATFPLSQVIPGWTEGVRLLKEGGEATFYIPSNLAYREQG-AGEKI 237

10_Ng DGTVFDSSKANGG-PATFPLSQVIPGWTEGVRLLKEGGEATFYIPSNLAYREQG-AGEKI 237

35_Ng DGTVFDSSKANGG-PATFPLSQVIPGWTEGVRLLKEGGEATFYIPSNLAYREQG-AGEKI 237

2_Nm DGTVFDSSKANGG-PVTFPLSQVIPGWTEGVQLLKEGGEATFYIPSNLAYREQG-AGDKI 237

13_Nm DGTVFDSSKANGG-TVTFPLSQVIPGWTEGVQLLKEGGEATFYIPSNLAYREQG-AGDKI 237

7_Nm DGTVFDSSKANGG-PVTFPLSQVIPGWTEGVQLLKEGGEATFYIPSNLAYREQG-AGDKI 233

1_Nm DGTVFDSSKANGG-PVTFPLSQVIPGWTEGVQLLKEGGEATFYIPSNLAYREQG-AGDKI 233

22_Nm DGTVFDSSKANGG-PVTFPLSQVIPGWTEGVQLLKEGGEATFYIPSNLAYREQG-AGDKI 233

44518_benedictiae DGTVFDSSKANGG-PATFPLSQVIPGWTEGVQLLKEGGEATFYIPSNLAYREQG-AGEKI 238

1770_lactamica DGTVFDSSKANGG-PATFPLSQVIPGWTEGVQLLKEGGEATFYIPSNLAYREQG-AGEKI 238

14730_polysaccharea DGTVFDSSKANGG-PATFPLSQVIPGWTEGVQLLKEGGEATFYIPSNLAYREQG-AGEKI 237

46231_blantyrii DGTVFDSSKANGG-PATFPLSQVIPGWTEGVQLLKEGGEATFYIPSNLAYREQG-AGEKI 237

43425_maigaei DGTVFDSSKANGG-PATFPLSQVIPGWTEGVQLLKEGGEATFYIPSNLAYREQG-AGEKI 237

46405_viridae DGTVFDSSKANGG-PATFPLSQVIPGWTEGVQLLKEGGEATFYIPSNLAYREQG-AGDKI 237

46192_basseii DGTVFDSSKANGG-PATFPLSQVIPGWTEGVQLLKEGGEATFYIPSNLAYREQG-AGEKI 237

42909_bergeri DGTVFDSSKANGG-PATFPLSQVIPGWTEGVQLLKEGGEATFYIPSNLAYREQG-AGEKI 237

43128_uirgultaei DGTVFDSSKANGG-PATFPLSQVIPGWTEGVQLLKEGGEATFYIPSNLAYREQG-AGDKI 237

********* **. .:**:.******:*.::*:********:**::*** : . * **

19091_oralis APNATLIFDVKLLKVGKPESMPAAPSAAEASGQKSQ 271

3565_mucosa GPNSTLVFDVKLVKIGKPEDF-QPAPGQVDI-KKVQ 273

43658_subflava GPNATLVFDVKLVKVGAPDAA-AQQPVQVDV-QKVQ 271

38978_cinerea GPNSTLVFDVKLVKVGAPENAPAQQPAQVDI-KKVN 273

140_Ng GPNATLVFDVKLVKIGAPENAPAKQPDQVDI-KKVN 272

10_Ng GPNATLVFDVKLVKIGAPENAPAKQPDQVDI-KKVN 272

35_Ng GPNATLVFDVKLVKIGAPENAPAKQPDQVDI-KKVN 272

2_Nm GPNATLVFDVKLVKIGAPENAPAKQPAQVDI-KKVN 272

13_Nm GPNATLVFDVKLVKIGAPENAPAKQPAQVDI-KKVN 272

7_Nm GPNATLVFDVKLVKIGAPENAPAKQPAQVDI-KKVN 268

1_Nm GPNATLVFDVKLVKIGAPENAPAKQPAQVDI-KKVN 268

22_Nm GPNATLVFDVKLVKIGAPENAPAKQPAQVDI-KKVN 268

44518_benedictiae GPNSTLVFDVKLVKVGAPGNASAQQPAQVDI-KKVN 273

1770_lactamica GPNATLVFDVKLVKVGAPENAPAKQPVQVDI-KKVN 273

14730_polysaccharea GPNSTLVFDVKLVKVGAPENASAQQPAQVDI-KKVN 272

46231_blantyrii GPNSTLVFDVKLVKVGAPENAPAQQPAQVDI-KKVN 272

43425_maigaei GPNSTLVFDVKLVKVGAPENAPAQQPAQVDI-KKVN 272

46405_viridae GPNSTLVFDVKLVKVGAPENAPAKQPVQVDI-KKVN 272

46192_basseii GPNSTLVFDVKLVKVGAPENASAQQPAQVEI-KKVN 272

42909_bergeri GPNSTLVFDVKLVKVGAPENAPAQQPAQVDI-KKVN 272

43128_uirgultaei GPNSTLVFDVKLVKVGAPENAPAKQPVQVDI-KKVN 272

.**:**:*****:*:* * :* :

*denotes identical amino acid

No label, . and : denote one or more amino acid differences. Boxed area in red denotes the N-terminal truncated region 22 – 143 used in MIP vaccines.
