# Supplementary material for: Update on the Neisseria Macrophage Infectivity Potentiator-Like PPIase Protein
Source: Front Cell Infect Microbiol. 2022 Mar 22;12:861489. doi: 10.3389/fcimb.2022.861489 (PMC8981591; doi:10.3389/fcimb.2022.861489)
Supplement: Supplementary file 9 [file Table_6.docx]

**Supplementary Table 1. Distribution of MIP alleles amongst gonococci and meningococci.**

| **Ng-MIP Allele** | **No. isolates** | **% of total isolates** | **Nm-MIP Allele** | **No. isolates** | **% of total isolates** |
| --- | --- | --- | --- | --- | --- |
| **10** (+ 139, 256, 138, 697, 403, 254, 402, 297, 558, 705, 448, 487, 484) | 8235 | 64.31 | **2** (+4, 12, 14, 25, 62, 73, 93, 97, 98, 99, 104, 118, 123, 124, 129, 150, 153, 156, 165, 167, 170, 171, 181, 183, 186, 192, 193, 194, 224, 232, 237, 239, 241, 242, 243, 245, 258, 261, 263, 264, 268, 286, 296, 300, 311, 312, 314, 352, 361, 364, 377, 400, 433, 437, 449, 460, 464, 470, 481, 485, 493, 501, 516, 518, 523, 524, 531, 534, 543, 544, 573, 582, 607, 620, 636, 639, 675, 683, 689, 709, 725, 731, 1/4) | 11912 | 44.22 |
| **35** (+408, 476, 447, 556, 210, 410, 412, 562, 690, 691) | 2866 | 22.38 | **1** (+108, 154, 158, 161, 172, 185, 195, 198, 199, 223, 267, 284, 303, 354, 369, 375, 450, 458, 459, 463, 469, 478, 488, 499, 509, 525, 548, 591, 629, 640, 741, 743, 434) | 7471 | 27.73 |
| **140** (+206, 560) | 696 | 5.44 | **7** (+ 68, 75, 169, 180, 220, 285, 423, 574, 600, 623, 650, 7/12) | 2616 | 9.71 |
| **8** +(34, 201, 401) | 457 | 3.57 | **22** (+57, 117, 126, 149, 187, 233, 238, 248, 341, 368, 381, 637, 641, 652, 31, 32, 65, 67, 70, 111, 121, 168, 221, 305, 407, 491, 728, 738, 740) | 1615 | 5.99 |
| **205** | 259 | 2.02 | **13** (+26, 76, 151, 277, 298, 373, 454, 492, 495, 515, 724, 730) | 1522 | 5.65 |
| **203** (+214) | 108 | 0.84 | **6** (+23, 30, 128, 218, 436, 496, 511, 519, 532, 684) | 606 | 2.25 |
| **202** (+212, 707) | 102 | 0.80 | **63** (+444, 404) | 361 | 1.34 |
| **208** (+694) | 33 | 0.26 | **5** (+115, 372, 452, 455, 514, 569, 609, 660) | 300 | 1.11 |
| **63** | 10 | 0.08 | **107** (+127) | 117 | 0.43 |
| **204** | 7 | 0.05 | **66** (+69, 475) | 80 | 0.30 |
| **209** (+213) | 7 | 0.05 | **11** (+122) | 29 | 0.11 |
| **559** (+561) | 3 | 0.02 | **166** (+302) | 24 | 0.09 |
| **477** | 2 | 0.02 | **109** | 19 | 0.07 |
| **557** | 2 | 0.02 | **116** | 13 | 0.05 |
| **200** | 1 | 0.01 | **141** | 13 | 0.05 |
| **563** | 1 | 0.01 | **110** (+190) | 11 | 0.04 |
| **225** | 1 | 0.01 | **216** (+497, 667) | 9 | 0.03 |
| **207** | 1 | 0.01 | **132** (+145, 244) | 7 | 0.03 |
| **695** | 1 | 0.01 | **94** (+247, 506) | 7 | 0.03 |
| **692** | 1 | 0.01 | **366** | 7 | 0.03 |
| **706** | 1 | 0.01 | **197** | 6 | 0.02 |
| **703** | 1 | 0.01 | **439** | 6 | 0.02 |
| **681** | 1 | 0.01 | **74** (+95) | 5 | 0.02 |
| **411** | 1 | 0.01 | **125** (+426) | 5 | 0.02 |
| **409** | 1 | 0.01 | **415** (+723) | 5 | 0.02 |
| **137** | 1 | 0.01 | **465** (+527, 567) | 5 | 0.02 |
| **605** | 1 | 0.01 | **105** | 5 | 0.02 |
| **693** | 1 | 0.01 | **112** (+246) | 4 | 0.01 |
| **585** | 1 | 0.01 | **507** (+510) | 4 | 0.01 |
| **56** | 1 | 0.01 | **657** | 4 | 0.01 |
| **722** | 1 | 0.01 | **215** | 4 | 0.01 |
| **211** | 1 | 0.01 | **27** (+656) | 4 | 0.01 |
|  |  |  | **500** | 3 | 0.01 |
|  |  |  | **320** | 3 | 0.01 |
|  |  |  | **502** | 3 | 0.01 |
|  |  |  | **196** | 3 | 0.01 |
|  |  |  | **494** | 3 | 0.01 |
|  |  |  | **10** | 3 | 0.01 |
|  |  |  | **35** | 3 | 0.01 |
|  |  |  | **9** | 2 | 0.01 |
|  |  |  | **655** | 2 | 0.01 |
|  |  |  | **438** | 2 | 0.01 |
|  |  |  | **148** | 2 | 0.01 |
|  |  |  | **365** | 2 | 0.01 |
|  |  |  | **182** | 2 | 0.01 |
|  |  |  | **498** | 2 | 0.01 |
|  |  |  | **179** | 2 | 0.01 |
|  |  |  | **625** | 2 | 0.01 |
|  |  |  | **708** | 2 | 0.01 |
|  |  |  | **287** | 2 | 0.01 |
|  |  |  | **638** | 2 | 0.01 |
|  |  |  | **217** | 2 | 0.01 |
|  |  |  | **229** | 2 | 0.01 |
|  |  |  | **15** | 1 | 0.00 |
|  |  |  | **58** | 1 | 0.00 |
|  |  |  | **658** | 1 | 0.00 |
|  |  |  | **374** | 1 | 0.00 |
|  |  |  | **541** | 1 | 0.00 |
|  |  |  | **687** | 1 | 0.00 |
|  |  |  | **324** | 1 | 0.00 |
|  |  |  | **262** | 1 | 0.00 |
|  |  |  | **295** | 1 | 0.00 |
|  |  |  | **356** | 1 | 0.00 |
|  |  |  | **653** | 1 | 0.00 |
|  |  |  | **323** | 1 | 0.00 |
|  |  |  | **96** | 1 | 0.00 |
|  |  |  | **257** | 1 | 0.00 |
|  |  |  | **399** | 1 | 0.00 |
|  |  |  | **528** | 1 | 0.00 |
|  |  |  | **405** | 1 | 0.00 |
|  |  |  | **622** | 1 | 0.00 |
|  |  |  | **299** | 1 | 0.00 |
|  |  |  | **688** | 1 | 0.00 |
|  |  |  | **321** | 1 | 0.00 |
|  |  |  | **147** | 1 | 0.00 |
|  |  |  | **716** | 1 | 0.00 |
|  |  |  | **570** | 1 | 0.00 |
|  |  |  | **483** | 1 | 0.00 |
|  |  |  | **406** | 1 | 0.00 |
|  |  |  | **396** | 1 | 0.00 |
|  |  |  | **331** | 1 | 0.00 |
|  |  |  | **729** | 1 | 0.00 |
|  |  |  | **726** | 1 | 0.00 |
|  |  |  | **540** | 1 | 0.00 |
|  |  |  | **572** | 1 | 0.00 |
|  |  |  | **146** | 1 | 0.00 |
|  |  |  | **290** | 1 | 0.00 |
|  |  |  | **371** | 1 | 0.00 |
|  |  |  | **627** | 1 | 0.00 |
|  |  |  | **659** | 1 | 0.00 |
|  |  |  | **114** | 1 | 0.00 |
|  |  |  | **155** | 1 | 0.00 |
|  |  |  | **265** | 1 | 0.00 |
|  |  |  | **219** | 1 | 0.00 |
|  |  |  | **367** | 1 | 0.00 |
|  |  |  | **370** | 1 | 0.00 |
|  |  |  | **440** | 1 | 0.00 |
|  |  |  | **466** | 1 | 0.00 |
|  |  |  | **480** | 1 | 0.00 |
|  |  |  | **486** | 1 | 0.00 |
|  |  |  | **517** | 1 | 0.00 |
|  |  |  | **521** | 1 | 0.00 |
|  |  |  | **554** | 1 | 0.00 |
|  |  |  | **571** | 1 | 0.00 |
|  |  |  | **608** | 1 | 0.00 |
|  |  |  | **686** | 1 | 0.00 |
|  |  |  | **727** | 1 | 0.00 |
|  |  |  | **453** | 1 | 0.00 |
|  |  |  | **535** | 1 | 0.00 |
|  |  |  | **152** | 1 | 0.00 |
|  |  |  | **240** | 1 | 0.00 |
|  |  |  | **3** | 1 | 0.00 |
|  |  |  | **628** | 1 | 0.00 |
|  |  |  | **568** | 1 | 0.00 |
|  |  |  | **512** | 1 | 0.00 |
|  |  |  | **736** | 1 | 0.00 |
|  |  |  | **654** | 1 | 0.00 |
|  |  |  | **441** | 1 | 0.00 |
|  |  |  | **294** | 1 | 0.00 |
|  |  |  | **157** | 1 | 0.00 |
|  |  |  | **435** | 1 | 0.00 |
|  |  |  | **630** | 1 | 0.00 |
|  |  |  | **301** | 1 | 0.00 |
|  |  |  | **685** | 1 | 0.00 |
|  |  |  | **545** | 1 | 0.00 |
|  |  |  | **266** | 1 | 0.00 |
|  |  |  | **710** | 1 | 0.00 |
|  |  |  | **526** | 1 | 0.00 |
|  |  |  | **680** | 1 | 0.00 |
|  |  |  | **589** | 1 | 0.00 |
|  |  |  | **490** | 1 | 0.00 |
|  |  |  | **419** | 1 | 0.00 |
|  |  |  | **259** | 1 | 0.00 |
|  |  |  | **189** | 1 | 0.00 |
|  |  |  | **106** | 1 | 0.00 |
|  |  |  | **222** | 1 | 0.00 |
|  |  |  | **188** | 1 | 0.00 |
|  |  |  | **553** | 1 | 0.00 |
|  |  |  | **604** | 1 | 0.00 |
|  |  |  | **130** | 1 | 0.00 |
|  |  |  | **549** | 1 | 0.00 |
|  |  |  | **359** | 1 | 0.00 |
|  |  |  | **322** | 1 | 0.00 |
|  |  |  | **24** | 1 | 0.00 |
|  |  |  | **113** | 1 | 0.00 |
|  |  |  | **717** | 1 | 0.00 |
|  |  |  | **319** | 1 | 0.00 |
|  |  |  | **88** | 1 | 0.00 |
|  |  |  | **529** | 1 | 0.00 |
|  | **Total = 12805** | **100%** |  | **Total = 26941** | **100%** |
