# Supplementary material for: Update on the Neisseria Macrophage Infectivity Potentiator-Like PPIase Protein
Source: Front Cell Infect Microbiol. 2022 Mar 22;12:861489. doi: 10.3389/fcimb.2022.861489 (PMC8981591; doi:10.3389/fcimb.2022.861489)
Supplement: Supplementary file 10 [file Table_7.docx]

**Supplementary Table 2. Analysis of Ng-MIP and Nm-MIP allele distribution for the most recent year with complete data**

| **Ng-MIP Allele** | **No. isolates** | **% of total isolates** | **Nm-MIP Allele** | **No. isolates** | **% of total isolates** |
| --- | --- | --- | --- | --- | --- |
| **10** (+ 139, 256) | 268 | 67 | **1** | 70 | 42 |
| **35** | 74 | 19 | **2** (+4, 12, 14, 314) | 67 | 40 |
| **140** | 42 | 10.5 | **22** (+31, 57) | 11 | 7 |
| **8** (=34,201) | 14 | 3.5 | **13** (+26, 373) | 5 | 3 |
| **203** | 2 | 0.5 | **5** | 4 | 2 |
|  |  |  | **68 (=7)** | 4 | 2 |
|  |  |  | **6** | 2 | 1 |
|  |  |  | **35** | 1 | 0.6 |
|  |  |  | **63** | 1 | 0.6 |
|  |  |  | **127 (=107)** | 1 | 0.6 |
|  | **Total = 400** | **Total =100%** |  | **Total =166** | **Total =100%** |
