# Supplementary material for: Update on the Neisseria Macrophage Infectivity Potentiator-Like PPIase Protein
Source: Front Cell Infect Microbiol. 2022 Mar 22;12:861489. doi: 10.3389/fcimb.2022.861489 (PMC8981591; doi:10.3389/fcimb.2022.861489)
Supplement: Supplementary file 11 [file Table_8.docx]

**Supplementary Table 3. Expression of MIP (NEIS1487) in other *Neisseria spp.***

| ***Neisseria spp.*** | **Total no. isolates in PubMLST/*Neisseria* database** | **NEIS1487 Allele** | **No. isolates per allele** | **Is allele present in gonococci?** | **Is allele present in meningococci?** |
| --- | --- | --- | --- | --- | --- |
| *animalis* | 1 | - | - | - | - |
| *animaloris* | 1 | - | - | - | - |
| *arctica* | 0 | - | - | - | - |
| *bacilliformis* | 4 | - | - | - | - |
| *basseii* | 2 | 474 | 2 | No | No |
| *benedictiae* | 3 | 79 | 3 | No | No |
| *bergeri* | 61 | 27 | 5 | No | Yes |
|  |  | 64 | 18 | No | No |
|  |  | 78 | 2 | No | No |
|  |  | 472 | 36 | No | No |
| *blantyrii* | 14 | 77 | 14 | No | No |
| *canis* | 2 | - | - | - | - |
| *cinerea* | 49 | 18 | 1 | No | No |
|  |  | 36 | 6 | No | No |
|  |  | 37 | 1 | No | No |
|  |  | 38 | 1 | No | No |
|  |  | 39 | 1 | No | No |
|  |  | 61 | 1 | No | No |
|  |  | 103 | 2 | No | No |
|  |  | 113 | 2 | No | Yes |
|  |  | 134 | 1 | No | No |
|  |  | 135 | 1 | No | No |
|  |  | 236 | 4 | No | No |
|  |  | 252 | 1 | No | No |
|  |  | 273 | 1 | No | No |
|  |  | 274 | 1 | No | No |
|  |  | 278 | 1 | No | No |
|  |  | 280 | 1 | No | No |
|  |  | 281 | 1 | No | No |
|  |  | 283 | 1 | No | No |
|  |  | 307 | 1 | No | No |
|  |  | 309 | 1 | No | No |
|  |  | 310 | 1 | No | No |
|  |  | 313 | 1 | No | No |
|  |  | 315 | 1 | No | No |
|  |  | 328 | 1 | No | No |
|  |  | 575 | 1 | No | No |
| *dentiae* | 1 | - | - | - | - |
| *dentrificans* | 0 | - | - | - | - |
| *dumasiana* | 0 | - | - | - | - |
| *elongata* | 2 | - | - | - | - |
| *elongata subsp elongate* | 1 | - | - | - | - |
| *elongata subsp. glycolytica* | 1 | - | - | - | - |
| *elongata subsp. nitroreducens* | 1 | - | - | - | - |
| *flava* | 0 | - | - | - | - |
| *flavescens* | 3 | - | - | - | - |
| *iguanae* | 1 | - | - | - | - |
| *lactamica* | 1317 | 55 | 240 | No | No |
|  |  | 58 | 85 | No | Yes |
|  |  | 15 | 52 | No | Yes |
|  |  | 260 | 39 | No | No |
|  |  | 9 | 24 | No | Yes |
|  |  | 184 | 24 | No | No |
|  |  | 100 | 21 | No | No |
|  |  | 162 | 20 | No | No |
|  |  | 101 | 17 | No | No |
|  |  | 16 | 12 | No | No |
|  |  | 83 | 11 | No | No |
|  |  | 379 | 10 | No | No |
|  |  | 59 | 6 | No | No |
|  |  | 330 | 4 | No | No |
|  |  | 351 | 4 | No | No |
|  |  | 271 | 3 | No | No |
|  |  | 430 | 3 | No | No |
|  |  | 71 | 2 | No | No |
|  |  | 136 | 2 | No | No |
|  |  | 324 | 2 | No | Yes |
|  |  | 334 | 2 | No | No |
|  |  | 357 | 2 | No | No |
|  |  | 586 | 2 | No | No |
|  |  | 595 | 2 | No | No |
|  |  | 90 | 1 | No | No |
|  |  | 163 | 1 | No | No |
|  |  | 164 | 1 | No | No |
|  |  | 276 | 1 | No | No |
|  |  | 343 | 1 | No | No |
|  |  | 350 | 1 | No | No |
|  |  | 355 | 1 | No | No |
|  |  | 358 | 1 | No | No |
|  |  | 380 | 1 | No | No |
|  |  | 382 | 1 | No | No |
|  |  | 421 | 1 | No | No |
|  |  | 422 | 1 | No | No |
|  |  | 424 | 1 | No | No |
|  |  | 425 | 1 | No | No |
|  |  | 427 | 1 | No | No |
|  |  | 428 | 1 | No | No |
|  |  | 429 | 1 | No | No |
|  |  | 431 | 1 | No | No |
|  |  | 432 | 1 | No | No |
|  |  | 442 | 1 | No | No |
|  |  | 457 | 1 | No | No |
|  |  | 566 | 1 | No | No |
|  |  | 583 | 1 | No | No |
|  |  | 587 | 1 | No | No |
|  |  | 596 | 1 | No | No |
| *macacae* | 1 | - | - | - | - |
| *maigaei* | 1 | 473 | 1 | No | No |
| *mucosa* | 33 | 21 | 1 | No | No |
|  |  | 28 | 3 | No | No |
|  |  | 29 | 1 | No | No |
|  |  | 33 | 1 | No | No |
|  |  | 43 | 1 | No | No |
|  |  | 49 | 1 | No | No |
|  |  | 50 | 1 | No | No |
|  |  | 52 | 2 | No | No |
|  |  | 60 | 1 | No | No |
|  |  | 82 | 1 | No | No |
|  |  | 84 | 1 | No | No |
|  |  | 87 | 1 | No | No |
|  |  | 131 | 1 | No | No |
|  |  | 269 | 1 | No | No |
|  |  | 270 | 1 | No | No |
|  |  | 395 | 1 | No | No |
|  |  | 711 | 2 | No | No |
| *mucosa subsp heidelbergensis* | 0 | - | - | - | - |
| *musculi* | 8 | - | - | - | - |
| *oralis* | 12 | 42 | 1 | No | No |
|  |  | 92 | 1 | No | No |
|  |  | 48 | 1 | No | No |
|  |  | 119 | 1 | No | No |
|  |  | 306 | 1 | No | No |
|  |  | 713 | 1 | No | No |
| *Perflava* | 4 | - | - | - | - |
| *Polysaccharea* | 79 | 17 | 15 | No | No |
|  |  | 41 | 1 | No | No |
|  |  | 80 | 1 | No | No |
|  |  | 86 | 8 | No | No |
|  |  | 88 | 3 | No | Yes |
|  |  | 89 | 3 | No | No |
|  |  | 229 | 6 | No | Yes |
|  |  | 253 | 1 | No | No |
|  |  | 271 | 1 | No | No |
|  |  | 272 | 1 | No | No |
|  |  | 279 | 5 | No | No |
|  |  | 345 | 1 | No | No |
|  |  | 385 | 1 | No | No |
|  |  | 392 | 1 | No | No |
|  |  | 398 | 2 | No | No |
|  |  | 416 | 13 | No | No |
|  |  | 471 | 1 | No | No |
|  |  | 555 | 1 | No | No |
| *shayeganii* | 1 | - | - | - | - |
| *sicca* | 2 | - | - | - | - |
| *skkuensis* | 0 | - | - | - | - |
| *subflava* | 79 | 318 | 5 | No | No |
|  |  | 19 | 3 | No | No |
|  |  | 44 | 2 | No | No |
|  |  | 54 | 2 | No | No |
|  |  | 85 | 2 | No | No |
|  |  | 120 | 2 | No | No |
|  |  | 325 | 2 | No | No |
|  |  | 353 | 2 | No | No |
|  |  | 376 | 2 | No | No |
|  |  | 394 | 2 | No | No |
|  |  | 584 | 2 | No | No |
|  |  | 20 | 1 | No | No |
|  |  | 40 | 1 | No | No |
|  |  | 45 | 1 | No | No |
|  |  | 72 | 1 | No | No |
|  |  | 91 | 1 | No | No |
|  |  | 102 | 1 | No | No |
|  |  | 133 | 1 | No | No |
|  |  | 178 | 1 | No | No |
|  |  | 282 | 1 | No | No |
|  |  | 289 | 1 | No | No |
|  |  | 291 | 1 | No | No |
|  |  | 308 | 1 | No | No |
|  |  | 316 | 1 | No | No |
|  |  | 326 | 1 | No | No |
|  |  | 327 | 1 | No | No |
|  |  | 329 | 1 | No | No |
|  |  | 360 | 1 | No | No |
|  |  | 378 | 1 | No | No |
|  |  | 383 | 1 | No | No |
|  |  | 417 | 1 | No | No |
|  |  | 451 | 1 | No | No |
|  |  | 456 | 1 | No | No |
|  |  | 712 | 1 | No | No |
|  |  | 714 | 1 | No | No |
|  |  | 718 | 1 | No | No |
| *tadorna* | 0 | - | - | - | - |
| *uirgultaei* | 9 | 362 | 9 | No | No |
| *Viridiae* | 21 | 81 | 21 | No | No |
| *Wadsworthii* | 1 | - | - | - | - |
| *Weaver* | 1 | - | - | - | - |
| *Zoodegmatis* | 2 | - | - | - | - |
| *Neisseria sp* | 64 | 10 | 1 | Yes | Yes |
|  |  | 86 | 1 | No | No |
| *non-Neisseria sp* | 3 | - | - | - | - |

|  |  |  |  |
| --- | --- | --- | --- |
|  |  |  |  |
|  |  |  |  |
|  |  |  |  |
|  |  |  |  |
|  |  |  |  |
|  |  |  |  |
|  |  |  |  |
|  |  |  |  |
|  |  |  |  |
|  |  |  |  |
|  |  |  |  |
|  |  |  |  |
|  |  |  |  |
|  |  |  |  |
